# Real‐World Health Care Resource Utilization and Costs Associated With First‐Line Dronedarone Versus First‐Line Ablation in Adults With Atrial Fibrillation

**DOI:** 10.1002/clc.70145

**Published:** 2025-05-29

**Authors:** Stephen J. Greene, Samantha Schilsky, Andrew W. Roberts, Shaum M. Kabadi, David S. McKindley, Ron Preblick, Jason Rashkin, Reno C. Leeming, Renee M. Sajedian, Andrea M. Russo

**Affiliations:** ^1^ Duke University School of Medicine Durham North Carolina USA; ^2^ Duke Clinical Research Institute Durham North Carolina USA; ^3^ Aetion Inc. New York New York USA; ^4^ Sanofi, Morristown New Jersey USA; ^5^ Cooper Medical School of Rowan University Camden New Jersey USA

**Keywords:** antiarrhythmic drugs, atrial fibrillation, catheter ablation, dronedarone, hospitalizations, recently diagnosed

## Abstract

**Background:**

Rhythm control therapy with antiarrhythmic drugs (AADs) or catheter ablation is recommended for treatment of atrial fibrillation (AF). The impact of first‐line AAD therapy (including dronedarone) or ablation on health care resource utilization (HCRU) is unclear.

**Methods:**

Optum's de‐identified Clinformatics Data Mart Database (January 1, 2012 to January 31, 2022) was used to assess US adults with AF (within 1 year) and no prior AADs who received first‐line dronedarone or first‐line ablation (including non‐dronedarone AADs then ablation within 90 days) using a comparative cohort design. Dronedarone and ablation cohorts were propensity score matched. HCRU and per‐patient per‐month (PPPM) payer costs were compared over 24‐months' follow‐up. Sensitivity analyses assessing first‐line ablation with no prior AADs were conducted.

**Results:**

Post‐matching, dronedarone and ablation cohorts (*n* = 1440) were similar. Event rate ratios (ERR; [95% CI]) for inpatient (0.85 [0.77–0.93]), any outpatient (0.95 [0.94–0.96]), or emergency room (0.91 [0.85–0.97]) visits, or atrial tachyarrhythmia (ATA)/AF–related procedures (0.72 [0.71–0.74]) were significantly lower with first‐line dronedarone versus ablation (all *p* < 0.01). Dronedarone was associated with reduced mean PPPM costs for total HCRU (−$2603), any outpatient visits (−$2401), and ATA/AF–related procedures (−$1880) versus ablation (all *p* < 0.01). In contrast to the primary analysis, sensitivity analyses showed no significant difference in ERR for all‐cause inpatient or any outpatient visits, but dronedarone remained associated with significantly lower mean PPPM total costs.

**Conclusion:**

Over 24‐months' follow‐up in patients with AF, first‐line dronedarone was associated with comparable rates of inpatient/outpatient visits, and lower total payer costs compared with an ablation‐based approach.

## Graphical abstract

A retrospective, observational, claims‐based cohort study identified that over 24‐months follow‐up of patients recently diagnosed with AF, a strategy of first‐line dronedarone was associated with similar or lower rates of any outpatient or inpatient visits, and lower total payer costs compared with an ablation‐based approach.

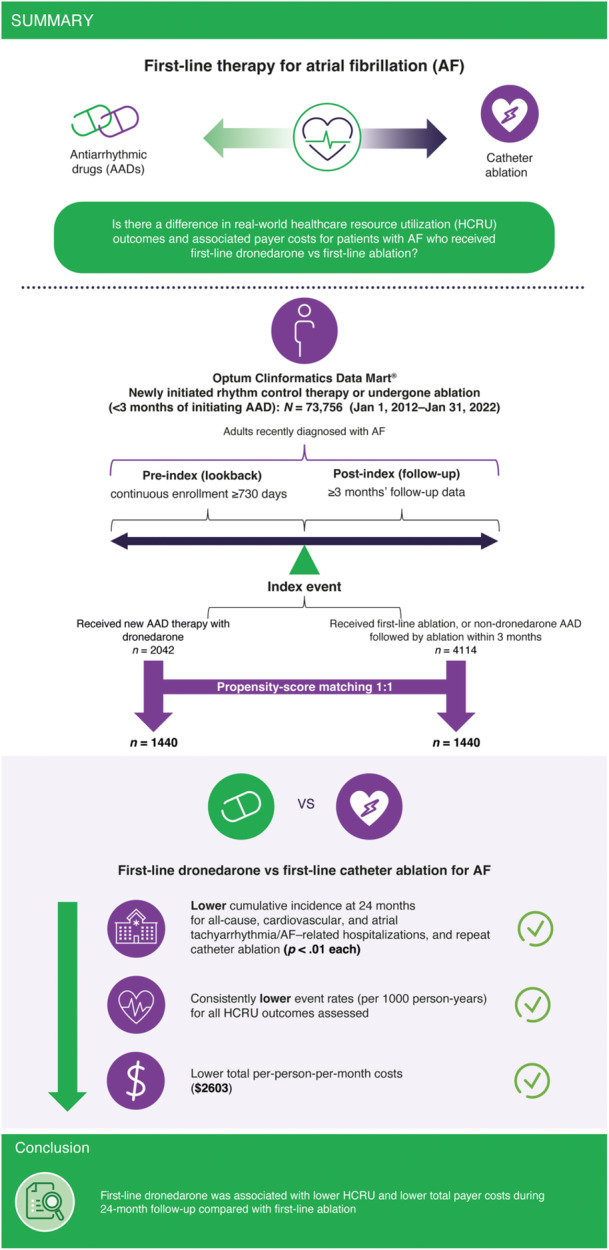

**Abbreviations**
AAD: antiarrhythmic drug
AF: atrial fibrillation
ATA: atrial tachyarrhythmia
CCI: Charlson Comorbidity Index
CV: cardiovascular
ER: emergency room
ERR: event rate ratio(s)
HCRU: health care resource utilization
*ICD‐9/10‐CM*
: *International Classification of Diseases, Ninth/Tenth Edition, Clinical Modification*
NYHA: New York Heart Association
PPPM: per‐patient per‐month
PSM: propensity score matching

## 1 Introduction

Atrial fibrillation (AF) is the most common sustained arrhythmia in the United States and worldwide [1, 2, 3]. The prevalence of AF in the United States is predicted to increase from approximately 5.2 million individuals in 2010 to ≥ 12.1 million in 2030 [4, 5]. Alongside this rising prevalence, AF is associated with substantial health care resource utilization (HCRU) and has a significant associated cost burden owing largely to its chronic nature [6, 7]. Based on US public and private health insurer records (1996–2016), HCRU costs associated with AF were estimated at $28 billion [5, 7].

Rhythm control therapy with antiarrhythmic drugs (AADs) or catheter ablation is recommended in the United States and European guidelines for patients with AF to improve symptoms and this may reduce hospitalizations, stroke, and mortality in patients with recently diagnosed AF (< 1 year) [8, 9, 10, 11]. In patients with AF and heart failure, rhythm control may also be useful in reducing mortality and heart failure–related hospitalizations [9]. Catheter ablation is supported as a first‐line approach in selected patients [9], based on evidence from studies such as the Catheter Ablation versus AAD Therapy for AF (CABANA) trial [12], to improve symptoms and reduce progression to persistent AF [9]. However, the health economic impact of an AAD or ablation approach is unclear [13].

With shared decision‐making, many patients may select AAD therapy over the more invasive approach of ablation. Consequently, a better understanding is needed of the real‐world HCRU and cost implications of adopting first‐line AAD therapy versus catheter ablation in patients recently diagnosed with AF. The appropriate AAD selection is based on the safety of the AAD for a particular patient and depends on comorbidities, presence or absence of underlying structural heart disease, coronary heart disease, New York Heart Association (NYHA) class, or presence of recent heart failure decompensation [9]. Studies of individual AADs are particularly relevant because amiodarone (the most frequently used AAD in the comparator group of CABANA) has been associated with long‐term toxicity and higher cardiovascular (CV) hospitalizations or death compared with rate control therapy alone [14]. In this context, the current study sought to assess all‐cause HCRU and associated payer costs in patients with recently diagnosed AF (within 1 year) receiving a rhythm control strategy of first‐line dronedarone therapy or first‐line ablation in the real‐world setting.

## 2 Methods

### 2.1 Data Source

**Figure 1:**
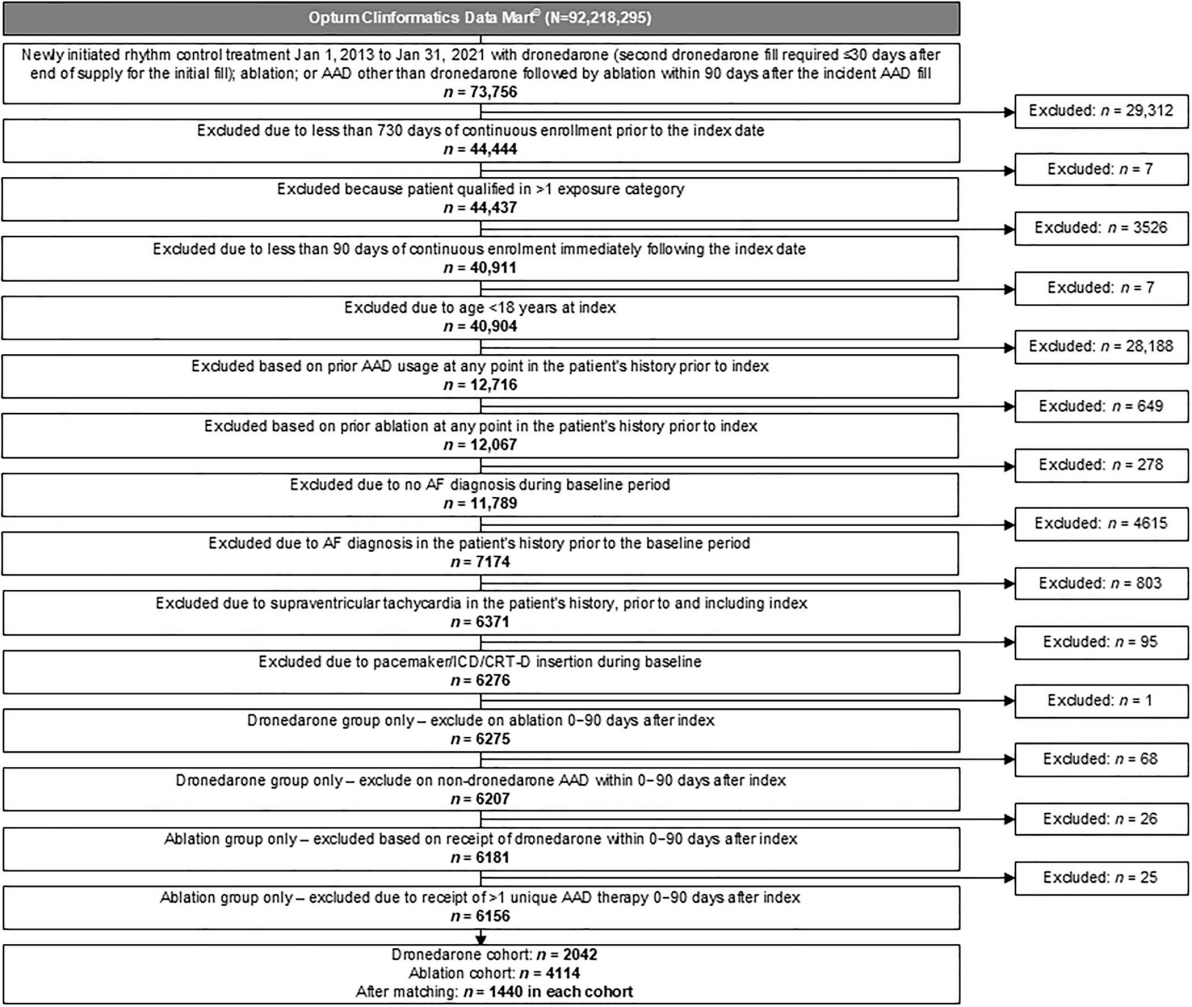
Study cohort selection flow diagram. AAD, antiarrhythmic drug; AF, atrial fibrillation; CRT‐D, cardiac resynchronization therapy with defibrillator; ICD, implantable cardioverter defibrillator.

This retrospective, observational cohort study used data from Optum's de‐identified Clinformatics Data Mart Database (CDM) between January 1, 2012 and January 31, 2022 (Supporting Information S1: Figure 1). CDM is derived from a database of administrative health claims for members of large commercial and Medicare Advantage health plans. CDM utilizes medical and pharmacy claims to derive patient‐level enrollment information, health care costs, and resource utilization information. The population is geographically diverse, spanning all 50 states and is statistically de‐identified under the Expert Determination method consistent with HIPAA and managed according to Optum's customer data use agreements. CDM administrative claims submitted for payment by providers and pharmacies are verified, adjudicated and de‐identified before inclusion.

### 2.2 Study Design and Populations

Adults aged ≥ 18 years with recently diagnosed AF between January 1, 2013 and January 31, 2021 and no prior AAD (amiodarone, sotalol, flecainide, propafenone, or dofetilide) exposure were identified. AF diagnosis was defined by *International Classification of Diseases*, *Ninth/Tenth Revision, Clinical Modification* (*ICD‐9/10‐CM*) codes for AF (Supporting Information S1: Table 1). Recent AF diagnosis was defined as the presence of an AF diagnosis during the baseline period spanning 365 days before index, with no AF diagnosis occurring in all available data before the baseline period. Patients were grouped by whether they subsequently received first‐line AAD therapy with dronedarone (“index” was the date of incident dronedarone prescription fill) or first‐line ablation (if they underwent an ablation procedure or received AAD therapy other than dronedarone followed by an ablation procedure within 90 days; “index” was the date of first recorded ablation procedure or non‐dronedarone AAD prescription fill) (Supporting Information S1: Figure 1). In the dronedarone cohort, patients were required to have at least 2 dronedarone prescription fills on separate days (with ≤ 30‐day gap between the first 2 prescription fills), and could not receive an ablation procedure within the first 90 days of therapy (to identify patients for whom maintenance of sinus rhythm with dronedarone alone was the aim of treatment, and for whom medication adherence was reasonable). Requiring incident ablation or ablation preceded by a brief trial of a single AAD was intended to capture patients in the first‐line ablation cohort for whom ablation was the intended treatment.

To better assess the robustness of results comparing strategies of first‐line dronedarone versus first‐line ablation, two sensitivity analyses were prespecified using modified comparator cohorts. The first sensitivity analysis was carried out among patients with ≥ 365 days of continuous enrollment post‐index. A second sensitivity analysis utilized a modified first‐line ablation cohort limited to patients with no prior AAD exposure at index (i.e., no AAD exposure in the 90 days preceding ablation). This second sensitivity analysis was included because it was unclear if patients with brief AAD exposure followed quickly by an ablation (as was allowed in the first‐line ablation cohort within the primary analysis) could inadvertently bias the ablation cohort towards a higher‐risk cohort that had “failed” initial AAD treatment.

Patients were excluded if they had < 730 days of continuous medical and pharmacy coverage enrollment before and including the index date or < 90 days of continuous enrollment post‐index. Patients were also excluded if they had a diagnosis of supraventricular tachycardia (before or at index) or history of pacemaker, implantable cardioverter defibrillator, or cardiac resynchronization therapy with defibrillator insertion during the baseline period. Patients could not receive any AAD or ablation procedure before index. In the first‐line dronedarone cohort, any patient who underwent an ablation procedure and/or had a non‐dronedarone AAD prescription fill on or within 90 days following the index dronedarone prescription fill were excluded. In the first‐line ablation cohort, patients with ≥ 1 additional non‐dronedarone AAD prescription fill within 90 days of index non‐dronedarone prescription fill were also excluded.

### 2.3 Study Measures

#### 2.3.1 Baseline Variables

Patient demographics were extracted at index. Clinical comorbidities and selected medications, including anticoagulants, rate control medications, and antihypertensives, were examined during the baseline period (Supporting Information S1: Table 2). CHA_2_DS_2_‐VASc and Charlson Comorbidity Index (CCI) scores were calculated during the baseline period and categorized as continuous or categorical (CHA_2_DS_2_‐VASc: low risk [score 0], intermediate risk [score 1], or high risk [scores 2–9]; CCI: 0, 1–2, 3–4, and ≥ 5), as described previously [15, 16]. Additional covariates adjusted for in the propensity score matching (PSM) are described below.

#### 2.3.2 Outcomes

All patients were followed for a minimum of 90 days and maximum of 24 months from index. Patients were censored at the earliest occurrence of the following: end of medical and/or pharmacy enrollment, end of the study period, end of follow‐up observation period, or death. All‐cause HCRU comprising inpatient hospital stays and any outpatient visits (including physician office visits or emergency room [ER] visits), any atrial tachyarrhythmia (ATA)/AF–related procedures and repeat catheter ablation (identified as described in Supporting Information S1: Table 2) during follow‐up were reported as annualized event rates per 1000 person‐years. Cumulative incidences for all‐cause hospitalization, CV‐related hospitalization, ATA/AF–related hospitalization, and repeat catheter ablation were assessed over a maximum of 24 months follow‐up based on *ICD‐9/10‐CM* and additional facility codes (Supporting Information S1: Table 2). CV‐related hospitalization was defined as an inpatient visit (hospital stay) with an *ICD‐9/10‐CM* diagnosis code for ATA (including AF), heart failure, acute coronary syndrome, stroke‐related conditions, and/or proarrhythmia (in the primary position). ATA/AF–related procedures were based on ≥ 1 of the following services: ablation, repeat catheter ablation, or cardioversion; implantable cardioverter defibrillator or cardiac resynchronization therapy with defibrillator insertion, or pacemaker implantation; and screening and monitoring procedures (eg, electrocardiogram, stress tests, implantable loop recorder, and other heart rhythm–monitoring procedures). An ablation (or repeat ablation) event was captured by any inpatient or ambulatory encounter using *Current Procedural Terminology* codes (Supporting Information S1: Table 2). In the ablation cohort, a repeat ablation was defined as any ablation procedure recorded on or after the index ablation. In the first‐line dronedarone cohort, patients had to first undergo an initial ablation during follow‐up followed by a second ablation to record a repeat ablation.

#### 2.3.3 PSM

Patients in the first‐line dronedarone cohort were matched (1:1) to patients in the first‐line ablation cohort (Supporting Information S1: Table 3). The propensity score model was adjusted for prespecified covariates, including select demographic characteristics (age, sex, region, payer type, health plan type, and year of index date), baseline clinical characteristics including CHA_2_DS_2_‐VASc clinical score components (congestive heart failure, hypertension, diabetes, prior stroke/transient ischemic attack/thromboembolism, and vascular disease [15]), additional CCI score components (dementia, chronic pulmonary disease, mild liver disease, moderate/severe liver disease, rheumatic disease, renal disease, peptic ulcer disease, any malignancy, metastatic solid tumor, HIV/AIDS, and paraplegia/hemiplegia [16]), obstructive sleep apnea, pulmonary hypertension, mitral stenosis, obesity, time from first AF diagnosis to index, time from start of all available data to index, atrial flutter during baseline, use of baseline therapy (i.e., cardioversion, direct‐acting oral anticoagulants, warfarin, P2Y12 agents, rate control medications, antihypertensives, and digoxin) and baseline all‐cause HCRU (inpatient days, any outpatient visits, ER visits).

All covariates were measured before initiation of exposure to avoid adjustment for potential mediators of the exposure‐outcome relationships under investigation [17]. Missing indicator variables were generated to capture missing or unknown values for any covariates to maximize patient inclusion. Model fit diagnostics were included to assess the absolute standardized difference (ASD) between cohorts for each matching factor. A covariate was considered balanced after PSM if it had an ASD ≤ 0.1 (10%) between cohorts [15]. For the first‐line dronedarone and first‐line ablation cohorts to be considered comparable after PSM, 90% of the covariates included within the propensity score model were required to have an ASD ≤ 0.1.

### 2.4 Statistical Analysis

Descriptive analyses of each HCRU outcome were reported for PSM cohorts and presented as *n* (%) for frequencies of patients with ≥ 1 HCRU event and mean ± SD for per‐patient count of total HCRU events. Categorical and continuous HCRU were compared between cohorts following PSM using the McNemar test [18] and paired *t* tests, respectively. Missing data was quantified in terms of the number of unique patients with missing data and was not imputed. Patients with missing data were assigned to unknown categories; means and medians only included patients with ≥ 1 record for the characteristic. Poisson regression models were used to estimate PSM–adjusted event rate ratios (ERRs) and 95% CI for HCRU count outcomes at 24 months of follow‐up.

Time to HCRU in PSM first‐line dronedarone and ablation cohorts for all‐cause hospitalization, CV‐related hospitalization, ATA/AF–related hospitalization, and repeat catheter ablation outcomes were compared by generating Kaplan‐Meier time‐to‐first recorded HCRU survival curves over a 24‐month follow‐up. Log‐rank tests were used to analyze differences between incidence survival curves for first‐line dronedarone and ablation cohorts.

All‐cause direct payer costs (mean, median) per‐patient per‐month (PPPM) were calculated on adjudicated claims starting at index as total payer costs incurred by a patient within a given follow‐up period divided by the total months of follow‐up for that patient. Payer‐paid portions were calculated by subtracting the patient cost (coinsurance plus copay plus deductible) from the standard cost per year in CDM for services incurred over the entirety of the HCRU event. Total months of observation time were calculated as days of post‐index follow‐up divided by 30. Total costs were calculated as the sum of inpatient and outpatient payer costs and were reported as PPPM costs. Generalized linear models were used to compare PPPM payer cost outcomes over 24 months of follow‐up between first‐line dronedarone and ablation cohorts. Generalized linear models were specified using a zero‐inflated negative binomial distribution or a negative binomial distribution and log link, as appropriate. All costs were adjusted to 2020 US dollars using inflation factors provided by CDM to allow direct comparison of costs between different years. Patients were rematched for each sensitivity analysis using the same PSM approach described for the 24‐month primary analysis. All analyses were conducted using the previously validated Aetion Evidence Platform (version R4.54.0.20220628) and R, version 3.4.2 [19]. No adjustments for multiple comparisons were conducted.

## 3 Results

### 3.1 Study Population and Characteristics

Among 73 756 patients who recently initiated rhythm control therapy with dronedarone or had undergone an ablation within 90 days of initiating an AAD other than dronedarone, 6156 met eligibility criteria (first‐line dronedarone *n* = 2042; first‐line ablation *n* = 4114) (Figure 1).

After PSM, the first‐line dronedarone (*n* = 1440) and first‐line ablation (*n* = 1440) cohorts were well balanced according to prespecified criteria (Table 1). Patients in the first‐line dronedarone and first‐line ablation cohorts were 68.4 and 68.1 years old at index, respectively, and were more commonly male (dronedarone: 57.7%, ablation: 57.0%). For both cohorts, the mean CCI score was 2.3. Mean CHA_2_DS_2_‐VASc score was 3.4 in the first‐line dronedarone cohort and 3.3 in the first‐line ablation cohort. Approximately 23% of each cohort had a history of heart failure (dronedarone: 23.6%, ablation: 23.1%).

**Table 1:** Select baseline^a^ demographics, characteristics, HCRU, and procedures for patients with AF before and after PSM for first‐line dronedarone and first‐line ablation cohorts.

	Unmatched cohort			PSM cohort		
	First‐line dronedarone (*n* = 2042)	First‐line ablation (*n* = 4114)	ASD	First‐line dronedarone (*n* = 1440)	First‐line ablation (*n* = 1440)	ASD^b^
Age at index,^c^ years, mean ± SD	70.2 ± 10.6	65.7 ± 10.6	0.43	68.4 ± 10.7	68.1 ± 9.4	0.03
*Age group at index date,* c *n (%)*			0.48			0.21
18–26	*n* ≤ 5	10 (0.2)		*n* ≤ 5	*n* ≤ 5	
27–44	32 (1.6)	156 (3.8)		32 (2.2)	28 (1.9)	
45–54	150 (7.3)	439 (10.7)		131 (9.1)	94 (6.5)	
55–64	353 (17.3)	1024 (24.9)		287 (19.9)	313 (21.7)	
65–74	727 (35.6)	1688 (41.0)		549 (38.1)	630 (43.8)	
75–84	656 (32.1)	754 (18.3)		386 (26.8)	354 (24.6)	
*Sex, n (%)* c			0.29			0.01
Male	1072 (52.5)	2729 (66.3)		831 (57.7)	821 (57.0)	
Female	970 (47.5)	1385 (33.7)		609 (42.3)	619 (43.0)	
*Region, n (%)* c			0.18			0.03
Midwest	403 (19.7)	1007 (24.5)		306 (21.3)	304 (21.1)	
South	1005 (49.2)	1757 (42.7)		667 (46.3)	679 (47.2)	
West	391 (19.1)	923 (22.4)		301 (20.9)	298 (20.7)	
Northeast	241 (11.8)	409 (9.9)		164 (11.4)	156 (10.8)	
Other/Unknown	2 (0.1)	18 (0.4)		2 (0.1)	3 (0.2)	
*Payer, n (%)* c			0.22			0.02
Commercial	664 (32.5)	1774 (43.1)		513 (35.6)	529 (36.7)	
Medicare Advantage	1378 (67.5)	2340 (56.9)		927 (64.4)	911 (63.3)	
Time from AF diagnosis to index date, days,^c^ mean ± SD	67.0 ± 88.7	115.8 ± 95.9	0.53	80.2 ± 96.5	84.6 ± 78.0	0.05
*Baseline comorbidities* a						
CHA_2_DS_2_‐VASc score,^c^, ^d^ mean ± SD	3.6 ± 1.8	3.0 ± 1.8	0.33	3.4 ± 1.8	3.3 ± 1.8	0.03
*CHA_2_DS_2_‐VASc category,* c, d *n (%)*			0.27			0.08
Low risk (score: 0)	53 (2.6)	313 (7.6)		49 (3.4)	68 (4.7)	
Intermediate risk (score: 1)	222 (10.9)	617 (15.0)		188 (13.1)	169 (11.7)	
High risk (score: 2–9)	1767 (86.5)	3184 (77.4)		1203 (83.5)	1203 (83.5)	
CCI,^c^, ^d^ mean ± SD	2.5 ± 2.3	2.1 ± 2.1	0.19	2.3 ± 2.2	2.3 ± 2.2	0.03
*CCI category,* c, d *n (%)*			0.18			0.07
0	415 (20.3)	1093 (26.6)		329 (22.8)	371 (25.8)	
1–2	787 (38.5)	1623 (39.5)		577 (40.1)	542 (37.6)	
3–4	457 (22.4)	820 (19.9)		305 (21.2)	293 (20.3)	
≥ 5	383 (18.8)	578 (14.0)		229 (15.9)	234 (16.3)	
*History of comorbidities, n (%)*						
Hypertension^c^	1745 (85.5)	3195 (77.7)	0.20	1193 (82.8)	1186 (82.4)	0.01
CAD	1601 (78.4)	3049 (74.1)	0.10	1064 (73.9)	1115 (77.4)	0.08
Vascular disease^c^	684 (33.5)	1519 (36.9)	0.07	490 (34.0)	485 (33.7)	0.01
Obesity^c^	608 (29.8)	1495 (36.3)	0.14	482 (33.5)	471 (32.7)	0.02
Diabetes^c^	637 (31.2)	1068 (26.0)	0.12	436 (30.3)	420 (29.2)	0.02
Heart failure^c^	464 (22.7)	1147 (27.9)	0.12	340 (23.6)	332 (23.1)	0.01
Hypothyroidism	495 (24.2)	781 (19.0)	0.13	332 (23.1)	308 (21.4)	0.04
Obstructive sleep apnea^c^	384 (18.8)	1121 (27.2)	0.20	319 (22.2)	314 (21.8)	0.01
CKD	488 (23.9)	808 (19.6)	0.10	298 (20.7)	297 (20.6)	0.00
COPD	413 (20.2)	531 (12.9)	0.20	250 (17.4)	223 (15.5)	0.05
Stroke, TIA, or TE^c^	305 (14.9)	399 (9.7)	0.16	169 (11.7)	170 (11.8)	0.00
Peripheral artery disease	214 (10.5)	266 (6.5)	0.15	140 (9.7)	94 (6.5)	0.12
Myocardial infarction	145 (7.1)	231 (5.6)	0.06	92 (6.4)	92 (6.4)	0.00
Pulmonary hypertension^c^	135 (6.6)	261 (6.3)	0.01	90 (6.3)	90 (6.3)	0.00
Venous thromboembolism	104 (5.1)	120 (2.9)	0.11	61 (4.2)	49 (3.4)	0.04
Hyperthyroidism	57 (2.8)	60 (1.5)	0.09	37 (2.6)	22 (1.5)	0.07
Mitral stenosis^c^	18 (0.9)	35 (0.9)	0.00	12 (0.8)	17 (1.2)	0.04
*Baseline procedures* a						
Cardioversion,^c^ *n* (%)	432 (21.2)	1652 (40.2)	0.42	383 (26.6)	394 (27.4)	0.02
ATA screening and monitoring, *n* (%)	2020 (98.9)	4104 (99.8)	0.10	1427 (99.1)	1437 (99.8)	0.09
*HCRU and costs*						
Inpatient days,^c^ mean ± SD	3.8 ± 7.4	2.6 ± 4.9	0.20	2.8 ± 5.7	2.9 ± 5.7	0.01
Any outpatient visits,^c^ mean ± SD	24.0 ± 22.4	24.3 ± 19.5	0.01	23.9 ± 21.9	24.5 ± 19.1	0.03
Outpatient—physician office visits,^c^ mean ± SD	9.8 ± 7.1	10.3 ± 6.5	0.07	10.1 ± 7.3	10.1 ± 6.3	0.01
Outpatient—ER visits,^c^ mean ± SD	1.3 ± 1.9	1.1 ± 1.5	0.13	1.2 ± 1.7	1.2 ± 1.7	0.02
Total medical and prescription payer costs,^e^$	33 528 ± 55 169	29 928 ± 44 294	0.07	31 411 ± 52 341	30 238 ± 51 718	0.02

*Note:* Sample counts less than *n* = 5 have been redacted to protect patient anonymity.

Abbreviations: AF, atrial fibrillation; ASD, absolute standardized difference; ATA, atrial tachyarrhythmia; CAD, coronary artery disease; CCI, Charlson Comorbidity Index; CKD, chronic kidney disease; COPD, chronic obstructive pulmonary disease; HCRU, health care resource utilization; PSM, propensity score matching; TE, thromboembolism; TIA, transient ischemic attack.

^a^ Demographic characteristics were assessed at index, baseline comorbidities and procedures were assessed during the baseline period, defined as 365 days before index;

^b^ A covariate was considered balanced after PSM if an ASD was ≤ 0.1 (10%) between cohorts;

^c^ Included in the propensity score model;

^d^ CHA_2_DS_2_‐VASc and CCI scores calculated during baseline period were both categorical and continuous. For PSM, patients were matched on individual components, and not on the scores themselves;

^e^ All costs were adjusted to 2020$ using the Standard Cost Amount field and Optum's Cost Factors so costs during the study period could be directly compared. Total costs were calculated as the sum of inpatient payer costs and any outpatient visit payer costs.

After PSM, the majority of patients in the first‐line dronedarone and first‐line ablation cohorts had been prescribed rate control medications (76.4% vs. 76.1%, respectively) and/or antihypertensives (62.6% vs. 60.7%, respectively), during the 1‐year baseline period (Table 2). Overall, 38.3% of the first‐line ablation cohort (*n* = 552) filled a non‐dronedarone AAD prescription in the 90 days before the ablation procedure, most commonly amiodarone (*n* = 246 [17.1%]) (Table 2).

**Table 2:** Medications filled during the baseline period for the unmatched and PSM cohorts of patients with AF treated with first‐line dronedarone or first‐line ablation.

Medication	Unmatched cohort			PSM cohort		
	First‐line dronedarone (*n* = 2042)	First‐line ablation (*n* = 4114)	ASD	First‐line dronedarone (*n* = 1440)	First‐line ablation (*n* = 1440)	ASD^b^
*AAD filled during baseline period* a						
DOACs^a^						
*Apixaban*, *n* (%)	518 (25.4)	1566 (38.1)	0.28	469 (32.6)	434 (30.1)	0.05
Daily dose, mean ± SD	9.5 ± 1.4	9.8 ± 0.9	0.24	9.6 ± 1.3	9.8 ± 1.1	0.12
*Rivaroxaban*, *n* (%)	292 (14.3)	763 (18.5)	0.12	234 (16.3)	242 (16.8)	0.02
Daily dose, mean ± SD	19.0 ± 3.0	19.7 ± 1.8	0.25	19.1 ± 2.9	19.7 ± 1.7	0.24
*Dabigatran*, *n* (%)	49 (2.4)	94 (2.3)	0.01	37 (2.6)	39 (2.7)	0.01
Daily dose, mean ± SD	293.5 ± 30.0	288.1 ± 43.7	0.14	291.4 ± 34.4	284.5 ± 48.7	0.16
*Edoxaban*, *n* (%)	*n* ≤ 5	0	0.04	*n* ≤ 5	0	0.05
Daily dose, mean ± SD	60.0 ± 0.0	—	—	60.0 ± 0.0	—	—
Any DOAC; *n* (%)^c^	844 (41.3)	2355 (57.2)	0.32	726 (50.4)	699 (48.5)	0.04
Warfarin, *n* (%)^a^, ^c^	192 (9.4)	293 (7.1)	0.08	128 (8.9)	136 (9.4)	0.02
Daily dose, mean ± SD	5.1 ± 1.9	5.0 ± 2.0	0.03	5.3 ± 2.0	4.9 ± 1.7	0.18
Digoxin, *n* (%)^a^, ^c^	111 (5.4)	250 (6.1)	0.03	87 (6.0)	79 (5.5)	0.02
P2Y12 agent, *n* (%)^a^, ^c^	232 (11.4)	286 (7.0)	0.15	124 (8.6)	126 (8.8)	0.01
Antihypertensives, *n* (%)^a^, ^c^						
ACE inhibitors	641 (31.4)	1095 (26.6)	0.11	423 (29.4)	434 (30.1)	0.02
ARB	557 (27.3)	909 (22.1)	0.12	379 (26.3)	372 (25.8)	0.01
Aldosterone	78 (3.8)	167 (4.1)	0.01	60 (4.2)	52 (3.6)	0.03
Diuretics	888 (43.5)	1403 (34.1)	0.19	610 (42.4)	583 (40.5)	0.04
Any antihypertensive	1346 (65.9)	2169 (52.7)	0.27	901 (62.6)	874 (60.7)	0.04
Rate control medications, *n* (%)^a^, ^c^						
Beta‐blockers	1295 (63.4)	2544 (61.8)	0.03	918 (63.7)	922 (64.0)	0.01
CCBs						
Non‐DHP CCBs	382 (18.7)	879 (21.4)	0.07	281 (19.5)	313 (21.7)	0.06
DHP‐CCBs	511 (25.0)	706 (17.2)	0.19	348 (24.2)	297 (20.6)	0.09
Any rate control medication	1563 (76.5)	2970 (72.2)	0.10	1100 (76.4)	1096 (76.1)	0.01
*AAD filled during index (ablation cohort only)*						
Amiodarone, *n* (%)	—	693 (16.8)		—	246 (17.1)	
Daily dose of index fill, mean ± SD	—	441.0 ± 261.3		—	445.3 ± 276.4	
Sotalol, *n* (%)	—	174 (4.2)		—	73 (5.1)	
Daily dose of index fill, mean ± SD	—	165.7 ± 55.9		—	161.1 ± 57.7	
Flecainide, *n* (%)	—	402 (9.8)		—	153 (10.6)	
Daily dose of index fill, mean ± SD	—	151.1 ± 60.3		—	145.2 ± 54.6	
Propafenone, *n* (%)	—	89 (2.2)		—	51 (3.5)	
Daily dose of index fill, mean ± SD	—	466.8 ± 121.4		—	466.2 ± 112.9	
Dofetilide, *n* (%)	—	70 (1.7)		—	29 (2.0)	
Daily dose of index fill, mean ± SD	—	785.5 ± 256.6		—	689.2 ± 264.7	
Any AAD, *n* (%)	—	1428 (34.7)		—	552 (38.3)	

*Note:* Sample counts less than *n* = 5 have been redacted to protect patient anonymity.

Abbreviations: AAD, antiarrhythmic drug; ACE, angiotensin converting enzyme; AF, atrial fibrillation; ARB, angiotensin receptor blocker; ASD, absolute standardized difference; CCB, calcium channel blocker; DHP, dihydropyridine; DOAC, direct‐acting oral anticoagulant; PSM, propensity score matching.

^a^ The baseline period was 365 days before index.

^b^ A covariate was considered balanced after PSM if an ASD was ≤0.1 (10%) between cohorts.

^c^ Included in the propensity score model (all variables are given in Supporting Information S1: Table 3).

## 4 HCRU

**Figure 2:**
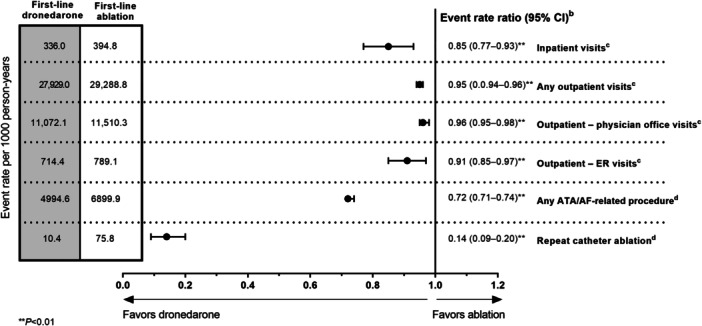
HCRU event rates and event rate ratios (95% CI) for patients with AF treated with first‐line dronedarone versus first‐line ablation at 24 months of follow‐up.^a^ ^a^Median follow‐up was 731 days in both first‐line dronedarone and ablation cohorts. ^b^Poisson regression models were used to estimate PSM‐adjusted event rates ratios and 95% CIs for HCRU count outcomes. ^c^All‐cause HCRU includes inpatient visits (hospital stay) and any outpatient visits (including outpatient physician office and ER visits). ^d^In the ablation cohort, a repeat ablation was defined as any ablation event recorded on or after index. In the dronedarone cohort, patients underwent ≥ 2 ablation procedures (as defined in the Methods). AF, atrial fibrillation; ATA, atrial tachyarrhythmia; ER, emergency room; HCRU, health care resource utilization; PSM, propensity score matching.

**Figure 3:**
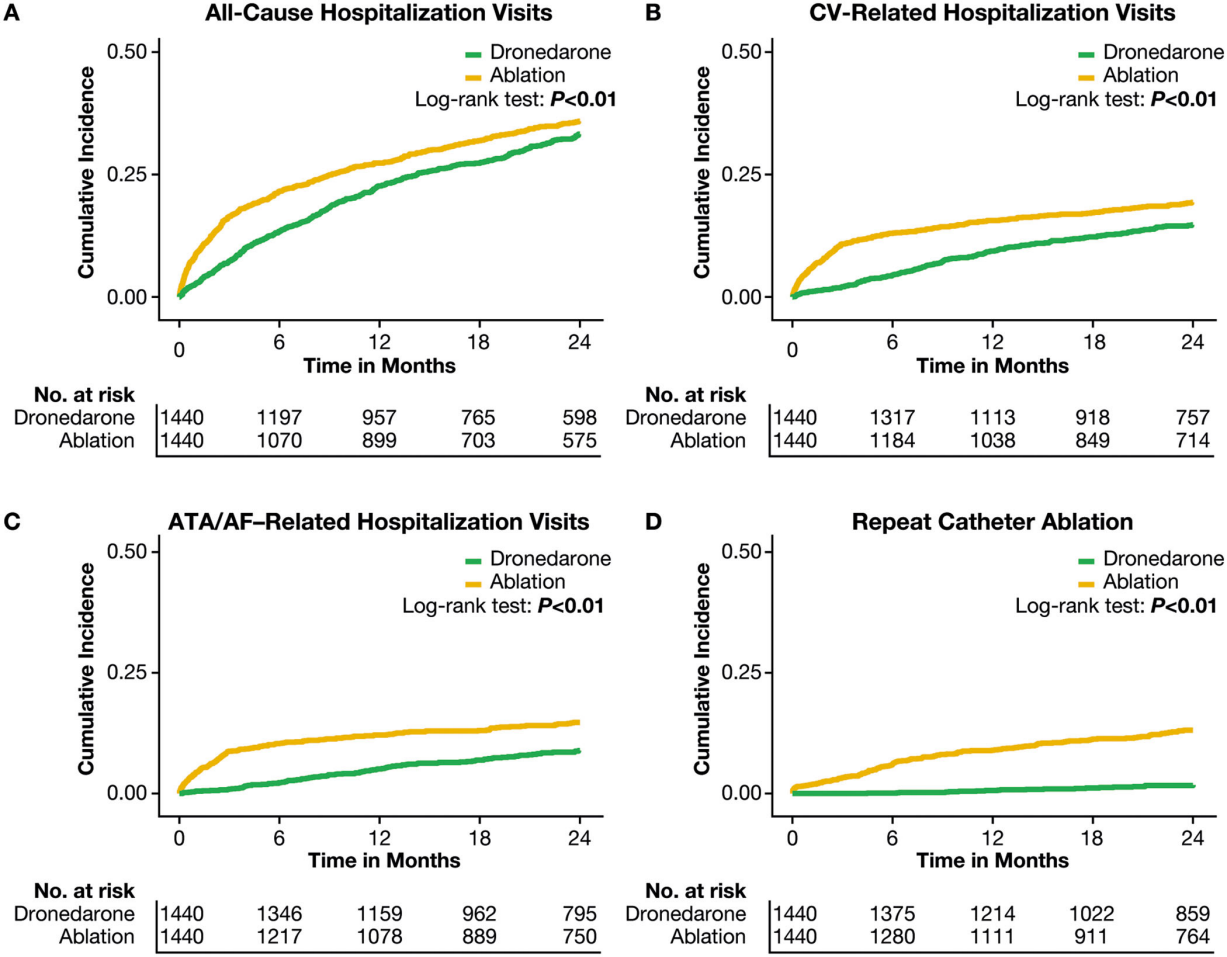
Cumulative incidence time‐to‐event over 24 months of follow‐up: (A) all‐cause hospitalization visits; (B) CV‐related hospitalizations; (C) ATA/AF–hospitalizations; (D) underwent ≥ 2 ablation procedures (repeat catheter ablation^a^). ^a^In the ablation cohort, a repeat ablation was defined as any ablation event recorded on or after index. In the dronedarone cohort, patients underwent ≥ 2 ablation procedures (as defined in the Methods). AF, atrial fibrillation; ATA, atrial tachyarrhythmia; CV, cardiovascular.

Median follow‐up time was 731 days (interquartile range: 493–731) in the first‐line dronedarone cohort and 731 days [495–731] in the first‐line ablation cohort. In the 90 days post‐index, 789 (54.8%) patients in the first‐line ablation cohort had filled a non‐dronedarone AAD prescription (Supporting Information S1: Table 4). At 24 months of follow‐up, event rates (per 1000 person‐years) were consistently lower with first‐line dronedarone versus ablation including for inpatient visits (0.85 [95% CI: 0.77–0.93]), any outpatient visits (0.95 [0.94–0.96]), including for physician office visits (0.96 [0.95–0.98]) and ER visits (0.91 [0.85–0.97]), and any ATA/AF–related procedures (0.72 [0.71–0.74]) (*p* < 0.01 for all; Figure 2). Cumulative incidence for time‐to‐first event was also lower with first‐line dronedarone compared with first‐line ablation for all‐cause hospitalizations, CV‐related hospitalizations, ATA/AF–related hospitalizations, and repeat catheter ablation (all *p* < 0.01; Figure 3). Over the 24‐month follow‐up, 11.6% (*n* = 167) of the first‐line ablation cohort and 1.4% (*n* = 20) of the dronedarone cohort underwent a repeat ablation procedure.

### 4.1 Payer Costs

**Figure 4:**
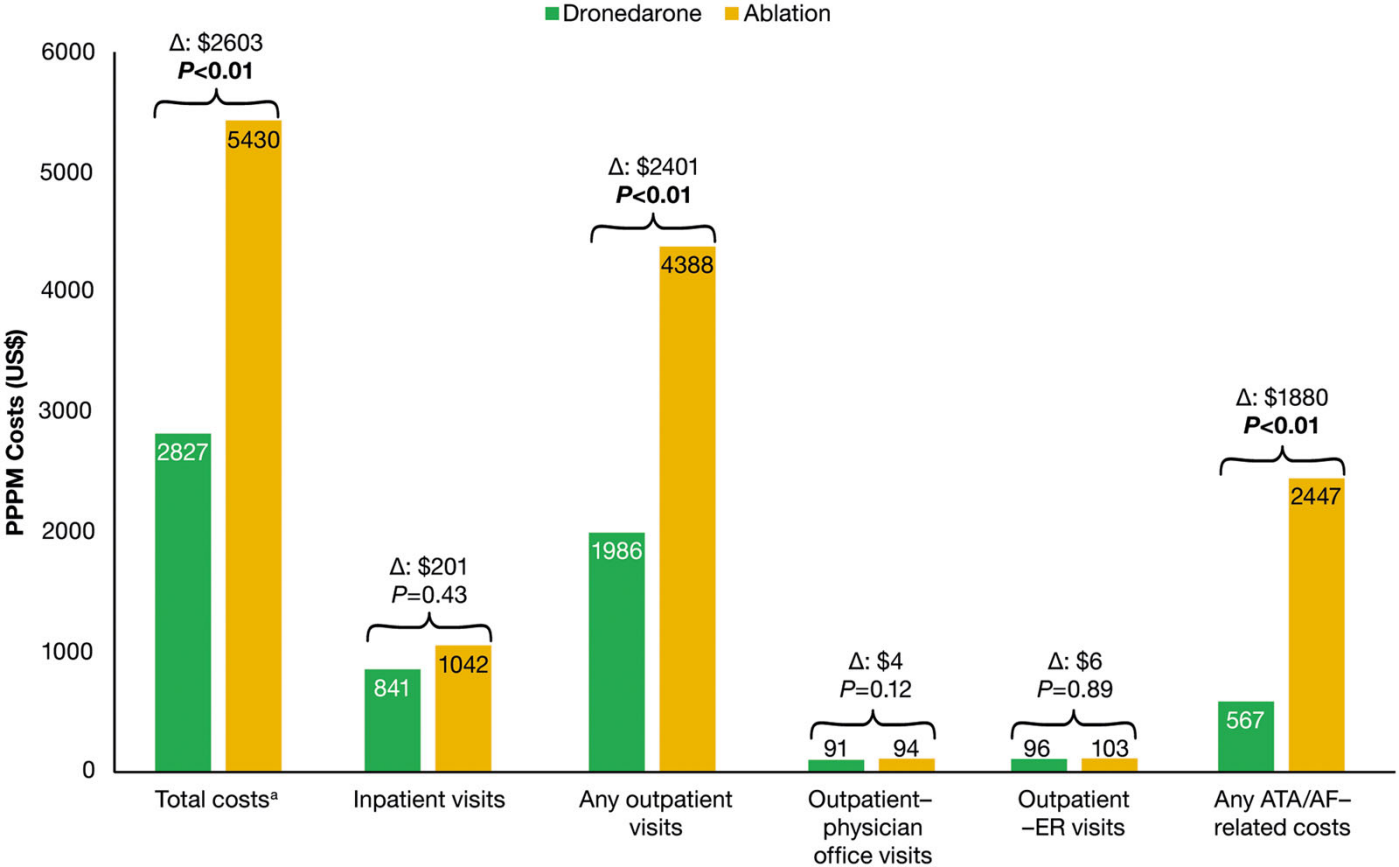
Annualized HCRU–associated PPPM costs for patients with AF treated with first‐line dronedarone versus first‐line ablation at 24 months of follow‐up. ^a^Mean total HCRU costs were calculated as the sum of inpatient payer costs and any outpatient visit payer costs (which included outpatient physician office visits and any ER visits). AF, atrial fibrillation; ATA, atrial tachyarrhythmia; CA, catheter ablation; CV, cardiovascular; ER, emergency room; HCRU, health care resource utilization; PPPM, per‐patient per‐month.

Total medical and prescription/payer costs during the baseline period were (mean ± SD) $31 411 ± 52 340 in the first‐line dronedarone cohort versus $30 238 ± 51 718 in the ablation cohort. Mean PPPM payer costs were generally lower with first‐line dronedarone compared with first‐line ablation at 24 months follow‐up (Figure 4). More specifically, total PPPM costs were $2603 lower with first‐line dronedarone, as were PPPM costs associated with any outpatient visits (−$2401) and ATA/AF–related procedures (−$1880) versus ablation cohort (all *p* < 0.01) (Figure 4).

### 4.2 Sensitivity Analysis of Patients With ≥ 12‐month Follow‐Up

In a sensitivity analysis of patients with ≥ 12 months of continuous post‐index enrollment (*n* = 1212 per cohort), observations were generally similar to the primary findings over the 24‐month follow‐up for HCRU (Supporting Information S1: Table 5) and PPPM costs (Supporting Information S1: Table 6). More specifically, first‐line dronedarone was associated with lower ERR for any inpatient and any outpatient, including physician office or ER visit (all *p* < 0.01; Supporting Information S1: Table 5), compared with first‐line ablation. First‐line dronedarone was associated with lower mean PPPM total costs versus ablation, and lower costs for outpatient and outpatient physician office visits (*p* < 0.01; Supporting Information S1: Table 6).

### 4.3 Sensitivity Analysis Defining First‐Line Ablation as Indexing on Ablation Only

After PSM, 1120 first‐line ablation patients underwent an ablation procedure without prescription for any AAD in the preceding 90 days. In both cohorts, mean age was 68.2 years and mean baseline CHA_2_DS_2_‐VASc score was 3.3; 83.6% of dronedarone and 82.7% of ablation‐only patients were in the “high risk” category (Supporting Information S1: Table 7). In contrast to the primary analysis, there was no difference between cohorts in the PSM–adjusted ERR for inpatient visits (ERR: 0.96 [95% CI: 0.86–1.08]; *p* = 0.52) or any outpatient visits (ERR: 1.00 [0.99–1.02]; *p* = 0.63) (Supporting Information S1: Table 8). The rate of all‐cause ER visits was 8% lower for first‐line dronedarone versus first‐line ablation patients indexing on ablation only (ERR: 0.92 [0.85–1.00]; *p* = 0.04). However, first‐line dronedarone patients had a 3% higher rate of physician office visits than patients indexing on ablation alone (ERR: 1.03 [1.01–1.05]; *p* < 0.01). For HCRU–associated payer costs, results were comparable with the primary analysis (Supporting Information S1: Table 9); mean PPPM total costs were lower for first‐line dronedarone than for first‐line ablation alone ($2968 vs. $5070, respectively). This resulted in an incremental total increase of +$2102 PPPM with first‐line ablation alone versus first‐line dronedarone.

## 5 Discussion

In this analysis of recently diagnosed patients with AF in United States clinical practice, dronedarone was associated with lower HCRU and lower total payer costs during 24 months of follow‐up compared with patients who underwent catheter ablation as part of a first‐line rhythm control strategy. The cumulative incidence for all‐cause, CV‐related, and ATA/AF–related hospitalizations was significantly lower in patients treated with dronedarone versus an ablation strategy, and event rates for inpatient visits, outpatient visits, and ATA/AF–related procedures were consistently lower. In contrast to the primary analysis, prespecified sensitivity analysis comparing patients who received an ablation without any pre‐ablation AAD exposure, dronedarone was not associated with a significant reduction in event rate for all‐cause inpatient visits or any outpatient visits. Dronedarone was associated with a small increase in rate of outpatient physician office visits. However, dronedarone remained associated with significantly lower mean total payer costs compared with patients who received an ablation without any pre‐ablation AAD exposure.

Efficacy studies have demonstrated the benefits of early rhythm control therapy for patients with recently diagnosed AF [20, 21, 22]. With catheter ablation being increasingly used (either alone or in combination with AADs) [8, 9, 10], the comparative HCRU and cost implications of achieving rhythm control based on an AAD or ablation strategy is of interest. The reduced rates of hospitalization associated with first‐line dronedarone in our primary analysis contrast with results from a prior meta‐analysis in which hospitalizations were 68% lower with an ablation approach compared with AADs as first‐line therapy for AF (5.6% vs. 18.7%; *p* < 0.001) [23]. However, direct comparison with the meta‐analysis may not be appropriate because it included studies published from 2005 onwards, and may not therefore reflect current clinical practice [24], and because most patients in the comparator arms of the trials received non‐dronedarone AADs [25, 26, 27].

In our cost analysis, compared with ablation, first‐line dronedarone was consistently associated with a significant reduction in total payer costs in recently diagnosed patients with AF over the follow‐up period. While differences in costs were most pronounced immediately after index, total payer costs remained significantly lower with dronedarone at 12 and 24 months. By capturing events and payer costs associated with index encounters, this study provides insights about health care resources in routine clinical practice. Some post‐index costs may relate to the required clinical supervision during administration of non‐dronedarone AADs. In addition, catheter ablation is increasingly being carried out in the outpatient setting, with associated reductions in costs compared with inpatient procedures [13, 28]. From the US‐payer perspective, a study comparing PPPM costs for dronedarone versus ablation or rate control medications with ablation found a cost savings for dronedarone over the 5‐year horizon [29]. Conversely, other cost‐effectiveness models suggested economic benefits for first‐line ablation over AAD therapy in terms of cost per quality‐adjusted life‐years gained, as the cost advantage for AADs reportedly lessens over 5 years of follow‐up [13, 28]. However, neither of these studies specifically assessed the cost‐effectiveness of dronedarone versus ablation, and use substantially different methodologies compared with the present study (the Reynolds et al 2009 study reported estimates from a 5‐year model; the Chew et al 2022 study assessed a lifetime model). Additionally, cost advantages of ablation should also consider the high rates of repeat ablation procedures, which can be up to 50% of the population over 5 years [30], with some patients going on to have ≥ 4 ablation procedures [31]. These patients are frequently underestimated or not captured in clinical trials assessing ablation, which often consider only the initial ablation procedure and costs. In our study, median follow‐up in both cohorts was 731 days, providing sufficient follow‐up for cost analyses to account for the skew in procedural outlay in the ablation cohort being encountered closer to index. Nevertheless, longer studies may be needed to determine economic outcomes, particularly in patients who require repeat (or multiple) ablation procedures and those who receive AADs after ablation [32, 33], to fully understand the long‐term cost implications of each approach. It is important to note that 54.8% of patients received AADs post‐ablation in this study, many of whom remained on AADs even after the 3‐month post‐index period. This 3‐month period would coincide with physicians prescribing during an empiric “blanking period” peri‐ or post‐ablation. Over the entire 24‐month follow‐up period, 58% of patients in the first‐line ablation cohort were prescribed an AAD (at some point) (Supporting Information S1: Table 4). This is in line with studies reporting that up to 51% of patients receive AADs following an ablation, and that a first‐line ablation strategy does not reliably obviate the need for the downstream AAD use [30, 34, 35, 36]. Therefore, the addition of long‐term AAD therapy following ablation procedure(s) would also drive up costs in the ablation group over time. These observations suggest that a “hybrid” approach of ablation plus AAD therapy may be utilized in real‐world practice for many patients.

In our study, treatment cohorts were designed to compare HCRU of first‐line rhythm control treatment pathways. The ablation cohort included patients who initiated rhythm control therapy with an ablation procedure or with a non‐dronedarone AAD followed by ablation within 90 days, to capture a scenario in which clinicians provide a brief trial of a single AAD in accordance with contemporary guidelines at that time [37, 38], before progressing to ablation. Although more contemporary guidelines are now available [9], during our study period, short‐term AAD therapy was often used in patients for symptomatic relief while awaiting procedural scheduling. This design also captured a scenario where physicians may use an AAD for a short period to “quiet down” AF before an intended ablation. Although it is possible that some patients within this cohort had “failed” on their AAD and subsequently progressed to an ablation, such treatment decisions would not be captured. Accordingly, we prespecified a sensitivity analysis limiting the first‐line ablation cohort to only patients who indexed on an ablation procedure without any prior AAD exposure in the preceding 90 days. In this sensitivity analysis, there was generally no difference in the rate of HCRU between dronedarone and first‐line ablation‐alone patients having received no prior AAD therapy, although rate of all‐cause ER visits was 8% lower for dronedarone versus first‐line ablation‐only patients.

### 5.1 Limitations

Limitations of this study should be noted. First, this study using the CDM may be considered geographically representative of the overall insured US population, but the generalizability to a wider population outside of the US or noninsured US individuals cannot be assumed. This study evaluated data over a 10‐year period, during which clinical practice guidelines, interventions, and ablation technology may have appreciably changed; however, index year was considered as a variable in our PSM model to attempt to mitigate this. Moreover, as with any claims‐based analysis, this study is limited by the accuracy of the electronic records. Second, all prescription claims are considered “filled” but may not have been taken, which would be more likely to impact the dronedarone cohort than the ablation cohort. Third, the type of ablation procedure is not recorded in the claims record, and therefore not available for analysis. Similarly, the ICD coding language used cannot reliably distinguish between paroxysmal and persistent AF subtypes; in previous studies, a high proportion of patients were reported to have codes for paroxysmal AF before and after codes for persistent AF [39]. Future studies may assess differences in HCRU and costs between these AF subtypes. Fourth, an immortal time bias may have been introduced by requiring patients to have a minimum period of treatment for entry into the cohort (e.g., ≥ 2 dronedarone prescription fills and ablation within 90 days of initiating non‐dronedarone AAD therapy in the first‐line ablation cohort). Patients who survived and continued rhythm control therapy for this immortal time period may not be representative of all patients who initiated incident rhythm control treatment with the study exposures. However, utilization of a 90‐day period for defining cohorts was applied consistently to both treatment cohorts, and thus may be an unlikely driver of any between‐group differences. Lastly, although cohorts were well‐matched on prespecified variables, observational analyses cannot prove causality and residual or unmeasured confounding may remain. For example, the decision to initiate patients on dronedarone versus another AAD or first‐line ablation is partially dependent on patient or provider preference (and on the prior experiences and knowledge of current guidelines of the provider), including patient preference for undergoing an ablation surgery, which cannot be measured within claims data. Some patients will not be candidates for ablation, which may also introduce bias if higher‐risk patients were more likely to receive AAD therapy than an ablation. In addition, some patients may not wish to take AADs; these inherent variabilities are present within observational studies of large patient populations, where patient preferences are naturally present and usually unaccounted for. While follow‐up was ≥24 months (median 731 days), it is possible that patients in the dronedarone cohort may have subsequently undergone ablation after the follow‐up period and that more patients in the ablation cohort may have subsequently received additional ablation procedures or initiated AAD therapy, all of which may impact on longer‐term costs, reflecting real‐life clinical practice. Finally, other factors, including symptoms, quality of life, and patient values and preferences, are not included in the current analysis but are likely to influence therapeutic choices.

## 6 Conclusion

In patients with recently diagnosed AF managed in routine clinical practice in the US, across primary and sensitivity analyses, first‐line dronedarone was associated with similar or lower rates of any inpatient or outpatient visits, and lower total payer costs, over 24 months of follow‐up compared with patients who underwent first‐line catheter ablation as part of a rhythm control strategy. Using administrative claims data from a large payer database, this real‐world study highlights the potential HCRU and economic benefits of first‐line dronedarone therapy as initial rhythm control therapy for AF. These hypothesis‐generating observations require validation in randomized controlled trials of patients with recently diagnosed AF.

## Ethics Statement

All data in Optum's de‐identified Clinformatics Data Mart Database are Health Insurance Portability and Accountability Act (HIPPA) (1996) compliant and de‐identified to adhere with all relevant US regulations and privacy laws. As such, this study only analyzed de‐identified data, which are a priori exempt from Institutional Review Board approval according to the Federal Policy for the Protection of Human Subjects “Common Rule” (1991, revised 2018).

## Conflicts of Interest

Stephen J. Greene has received research support from the Duke University Department of Medicine Chair's Research Award, American Heart Association, National Heart Lung and Blood Institute, Amgen, AstraZeneca, Boehringer Ingelheim, Bristol Myers Squibb, Cytokinetics, Merck & Co. Inc., Novartis, Pfizer, and Sanofi; has served on advisory boards for Amgen, AstraZeneca, Boehringer Ingelheim/Lilly, Bristol Myers Squibb, Cytokinetics, Roche Diagnostics, and Sanofi; serves as a consultant for Amgen, Bayer, Bristol Myers Squibb, Corteria Pharmaceuticals, CSL Vifor, Lexicon, Merck, PharmaIN, Roche Diagnostics, Sanofi, scPharmaceuticals, Tricog Health, Urovant Pharmaceuticals; and has received speaker fees from Bayer, Boehringer Ingelheim, Cytokinetics, Lexicon, and Roche Diagnostics. Samantha Schilsky, Andrew W. Roberts, Reno C. Leeming, and Renee M. Sajedian are employees of Aetion, which received funding for this analysis from Sanofi. Shaum Kabadi, David S. McKindley, and Ron Preblick are employees of Sanofi and may hold shares and/or stock options in the company. Jason Rashkin was an employee of Sanofi at the time of the study and may hold shares and/or stock options in the company. Andrea M. Russo reports research grants from Abbott, Bayer, Boston Scientific, and Medtronic; consulting fees or honoraria from Abbott, Atricure, Bayer, Biosense Webster, Biotronik, BMS‐Pfizer, Boston Scientific, Medtronic, PaceMate and Sanofi (but did not receive honoraria specific to this manuscript).

## Supporting information

Greene 554 ms ClinCardiol SUPPLEMENT 25Oct24 (2) clean.

## Data Availability

Qualified researchers may request access to data. Further details on Sanofi's data sharing criteria, eligible studies, and process for requesting access can be found at:  https://www.vivli.org/.
