# Supplementary material for: Real‐World Health Care Resource Utilization and Costs Associated With First‐Line Dronedarone Versus First‐Line Ablation in Adults With Atrial Fibrillation
Source: Clin Cardiol. 2025 May 29;48(6):e70145. doi: 10.1002/clc.70145 (PMC12120900; doi:10.1002/clc.70145)
Supplement: Supplementary file 1 — Greene 554 ms ClinCardiol SUPPLEMENT 25Oct24 (2) clean. [file CLC-48-e70145-s001.docx]

**Real-world healthcare resource utilization and costs associated with first-line dronedarone versus first-line ablation in adults with atrial fibrillation**

**Short title**: HCRU with first-line dronedarone vs ablation

**Stephen J. Greene MD^1,2^** [**stephen.greene@duke.edu**](mailto:stephen.greene@duke.edu) **| Samantha Schilsky PhD, MPH^3^** [**samantha.schilsky@aetion.com**](mailto:samantha.schilsky@aetion.com) **| Andrew W. Roberts PharmD, PhD^3^** [**drew.roberts@aetion.com**](mailto:drew.roberts@aetion.com) **| Shaum M. Kabadi PhD, MPH^4^** [**Shaum.Kabadi@sanofi.com**](mailto:Shaum.Kabadi@sanofi.com) **| David S. McKindley PharmD^4^** [**David.McKindley@sanofi.com**](mailto:David.McKindley@sanofi.com) **| Ron Preblick PharmD, MPH^4^** [**Ronald.Preblick@sanofi.com**](mailto:Ronald.Preblick@sanofi.com) **| Jason Rashkin MD^4*^ pathema72@gmail.com | Reno C. Leeming PhD, MS^3^** [**reno.leeming@aetion.com**](mailto:reno.leeming@aetion.com) **| Renee M. Sajedian BS^3^** [**Renee.Sajedian@aetion.com**](mailto:Renee.Sajedian@aetion.com) **| Andrea M. Russo MD^5^** [**russo-andrea@CooperHealth.edu**](mailto:russo-andrea@CooperHealth.edu)

^1^Duke University School of Medicine, Durham, NC, USA

^2^Duke Clinical Research Institute, Durham, NC, USA

^3^Aetion, Inc., New York, NY, USA

^4^Sanofi, Bridgewater, NJ, USA (*at the time of the study)

^5^Cooper Medical School of Rowan University, Camden, NJ, USA

**FIGURE S1 Study design schematic.**


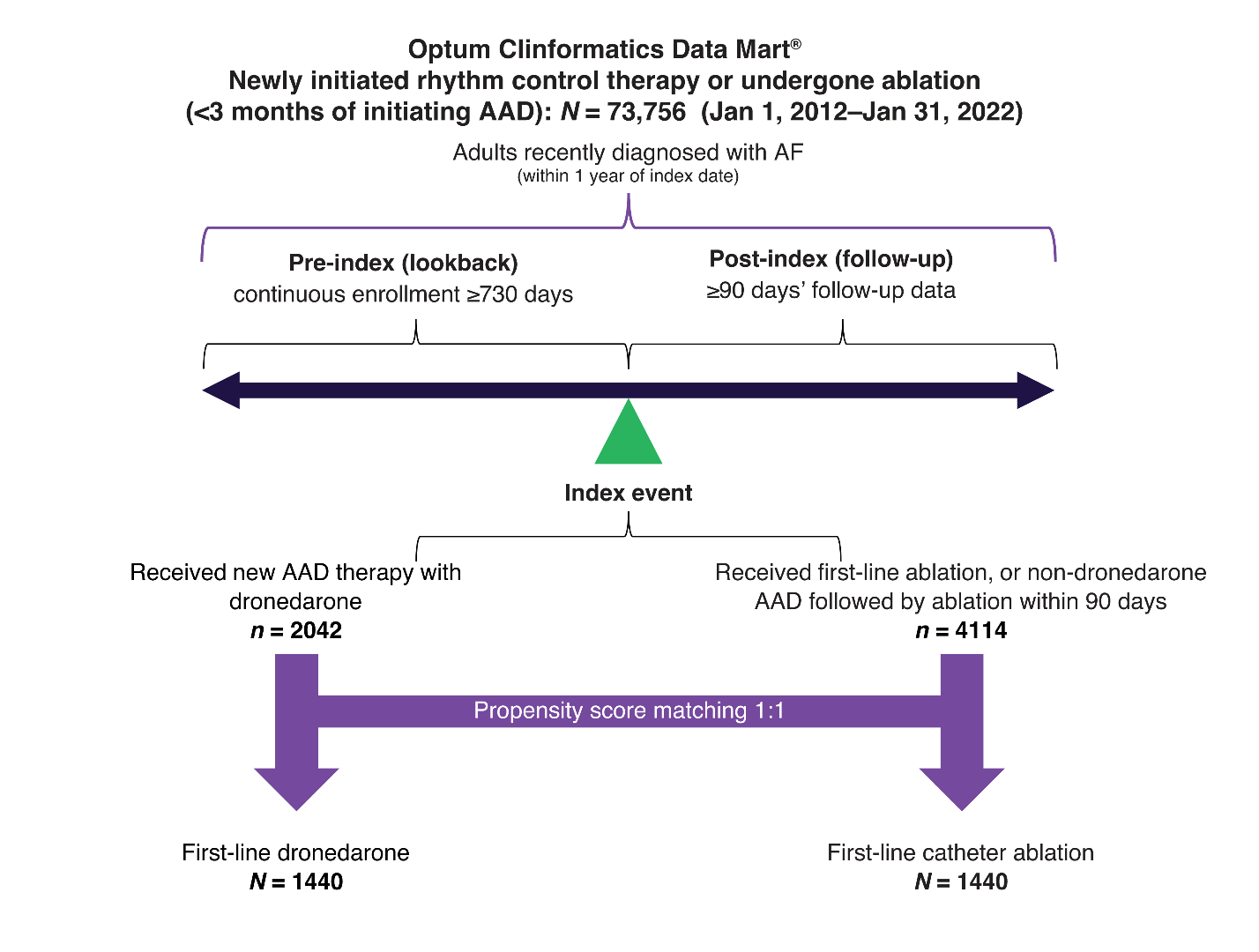


AAD, antiarrhythmic drugs; AF, atrial fibrillation.

**TABLE S1 Definitions of inclusion and exclusion criteria.**

| **Variable** | ***ICD-9* definition** | ***ICD-10* definition** |
| --- | --- | --- |
| Atrial fibrillation (AF) | Any inpatient or ambulatory encounter with an *ICD-9-CM* diagnosis code of 427.31 measured in any diagnosis position (ie, primary, secondary) | Any inpatient or ambulatory encounter with an *ICD-10-CM* diagnosis code of I48.0, I48.1* I48.2*, or I48.91 measured in any diagnosis position (ie, primary, secondary). |
| AADs other than dronedarone | The occurrence of any prescription claim with the following generic names: “Amiodarone,” “Sotalol,” “Flecainide,” “Propafenone,” or “Dofetilide” | |
| Catheter ablation | Any inpatient or ambulatory encounter with any of the following procedure codes in any claim line position:  *CPT*: 93656, 93657 | |
| Dronedarone | The occurrence of any prescription claim with the generic name “Dronedarone” | |
| ICD/CRT-D insertion | Any inpatient or ambulatory encounter with ≥1 of the following procedure codes in any claim line position:  *ICD-9-CM*: 37.94, 00.51, 00.54  *CPT/HCPCS:* 33230, 33231, 33240, 33249, 33270 | Any inpatient or ambulatory encounter with ≥1 of the following procedure codes in any claim line position:  *ICD-10-CM:* 0JH608Z, 0JH609Z, 0JH638Z, 0JH808Z, 0JH838Z, 0JH639Z, 0JH809Z, 0JH839Z  *CPT/HCPCS:* 33230, 33231, 33240, 33249, 33270 |
| Pacemaker implantation | Any inpatient or ambulatory encounter with ≥1 of the following procedure codes in any claim line position:  *ICD-9-CM:* 00.50, 00.52, 00.53, 37.78, 37.81, 37.82, 37.83  *CPT/HCPCS:* 0387T, 33206, 33207, 33208, 33212, 33213, 33214, 33221, 33227 | Any inpatient or ambulatory encounter with ≥1 of the following procedure codes in any claim line position:  *ICD-10-CM:* 0JH604Z, 0JH605Z, 0JH606Z,  0JH607Z, 0JH634Z, 0JH635Z, 0JH636Z, 0JH637Z, 0JH63PZ, 0JH804Z, 0JH805Z, 0JH806Z, 0JH807Z, 0JH80PZ, 0JH834Z, 0JH835Z, 0JH836Z, 0JH837Z, 0JH83PZ, 0JH605Z  *CPT/HCPCS:* 0387T, 33206, 33207, 33208, 33212, 33213, 33214, 33221, 33227 |
| Supraventricular tachycardia | Any inpatient or ambulatory encounter with an *ICD-9-CM* diagnosis code of 427.0 measured in any diagnosis position (ie, primary, secondary) | Any inpatient or ambulatory encounter with an *ICD-10-CM* diagnosis code of I47.1 measured in any diagnosis position (ie, primary, secondary). |
| AAD, antiarrhythmic drug; AF, atrial fibrillation; *CPT*, *Current Procedural Terminology*; CRT-D, cardiac resynchronization therapy with defibrillator; *HCPCS, Healthcare Common Procedure Coding System*; ICD, implantable cardioverter defibrillator; *ICD-9-CM*, *International Classification of Diseases, Ninth Revision, Clinical Modification*; *ICD-10-CM*, *International Classification of Diseases, Tenth Revision, Clinical Modification.* | | |

**TABLE S2 Medication and healthcare resource utilization variable definitions.**

| **Variable** | ***ICD-9* definition** | ***ICD-10* definition** |
| --- | --- | --- |
| Inpatient hospital stay (all-cause) | Any inpatient encounter where Type of Service is “FAC_IP.ACUTE” | |
| Outpatient visit – any | All outpatient encounter where Type of Service is “ANC.DMESRV”, “ANC.DRUGAD”, “ANC.HH/HPC”, “ANC.TRANSP”, “PROF.ALLERG”, “PROF.ANESTH”, “PROF.CELTHR”, “PROF.CONSUL”, “PROF.DENPRO”, “PROF.DIAGTS”, “PROF.IMMINJ”, “PROF.LAB”, “PROF.MH”, “PROF.OBSTET”, “PROF.PATHOL”, “PROF.PHYMED”, “PROF.PROOTH”, “PROF.PRVMED”, “PROF.RAD”, “PROF.SURG”, “PROF.VSHRSP”, “FAC_OP.FO_DIA”, “FAC_OP.FO_LAB”, “FAC_OP.FO_OTH”, “FAC_OP.FO_RAD”, “FAC_OP.FO_SUR”, “FAC_OP.ED”, “FAC_OP.ER”, PROF.ED”, “PROF.ER”, “PROF.OFFVIS”, “FAC_IP.ACUTE” or “FAC_IP.REHSNF” | |
| Outpatient visit – physician office  (all-cause) | Any outpatient encounter where Type of Service is “PROF.OFFVIS” | |
| Outpatient – emergency room visit (all-cause) | Any outpatient encounter where Type of Service is “PROF.ED,” “FAC_OP.ED,” “PROF.ER,” or “FAC_OP.ER” | |
| Cardiovascular-related hospitalization | Defined by its components below (atrial tachyarrhythmia hospitalization, heart failure hospitalization, acute coronary syndrome hospitalization, stroke-related hospitalization, proarrhythmia hospitalization) | |
| Atrial tachyarrhythmia hospitalization (including AF) | Any inpatient encounter with an *ICD-9-CM* diagnosis code of 427.0, 427.31, or 427.32 measured in the primary diagnosis position | Any inpatient encounter with an *ICD-10-CM* diagnosis code of I47.1, I48.3, 148.4, I48.0, I48.1* I48.2*, I48.91, or I48.92 measured in the primary diagnosis position |
| Heart failure hospitalization | Any inpatient encounter with ≥1 of the following *ICD-9-CM* diagnosis codes in the primary diagnosis position:  402.01, 402.11, 402.91, 404.01, 404.03, 404.11, 404.13, 404.91, 404.93, 428, 428.0, 428.1, 428.2, 428.20, 428.21, 428.22, 428.23, 428.3, 428.30, 428.31, 428.32, 428.33, 428.4, 428.40, 428.41, 428.42, 428.43, 428.9 OR the *ICD-9-PCS* code 37.66 in the principal position | Any inpatient encounter with ≥1 of the following *ICD-10-CM* diagnosis codes in the primary diagnosis position:  I11.0, I13.0, I13.2, I50.1, I50.20, I50.21, I50.22, I50.23, I50.30, I50.31, I50.32, I50.33, I50.40, I50.41, I50.42, I50.43, I50.810, I50.811, I50.812, I50.813, I50.814, I50.82, I50.83, I50.84, I50.89, I50.9 OR ≥1 of the following *ICD-10-PCS* codes in the principal position: 02HA0QZ, 02HA3QZ, 02HA4QZ |
| Acute coronary syndrome hospitalization | Any inpatient encounter with an *ICD-9-CM* diagnosis code of 410* or 411* measured in the primary diagnosis position | Any inpatient encounter with an *ICD-10-CM* diagnosis code of I21* or I22* measured in the primary diagnosis position |
| Stroke, TIA or TE-related hospitalization | Any inpatient encounter with ≥1 of the following codes in the primary diagnosis position:  *ICD-9-CM:* 415.1, 415.11, 415.12, 415.13, 415.19, 430, 431, 433.01, 433.11, 433.21, 433.31, 433.81, 433.91, 434.01, 434.11, 434.91, 435, 435.0, 435.1, 435.2, 435.3, 435.8, 435.9, 436, 444, 444.0, 444.01, 444.09, 444.1, 444.2, 444.21, 444.22, 444.8, 444.81, 444.89, 444.9 | Any inpatient encounter with ≥1 of the following codes in the primary diagnosis position:  *ICD-10-CM:* G45.0, G45.1, G45.2, G45.8, G45.9, G46.0, G46.1, G46.2, I26.01, I26.02, I26.09, I26.90, I26.92, I26.99, I60.00, I60.01, I60.02, I60.10, I60.11, I60.12, I60.2, I60.30, I60.31, I60.32, I60.4, I60.50, I60.51, I60.52, I60.6, I60.7, I60.8, I60.9, I61.0, I61.1, I61.2, I61.3, I61.4, I61.5, I61.6, I61.8, I61.9, I63.00, I63.011, I63.012, I63.013, I63.019, I63.02, I63.031, I63.032, I63.033, I63.039, I63.09, I63.10, I63.111, I63.112, I63.113, I63.119, I63.12, I63.131, I63.132, I63.133, I63.139, I63.19, I63.20, I63.211, I63.212, I63.213, I63.219, I63.22, I63.231, I63.232, I63.233, I63.239, I63.29, I63.30, I63.311, I63.312, I63.313, I63.319, I63.321, I63.322, I63.323, I63.329, I63.331, I63.332, I63.333, I63.339, I63.341, I63.342, I63.343, I63.349, I63.39, I63.40, I63.411, I63.412, I63.413, I63.419, I63.421, I63.422, I63.423, I63.429, I63.431, I63.432, I63.433, I63.439, I63.441, I63.442, I63.443, I63.449, I63.49, I63.50, I63.511, I63.512, I63.513, I63.519, I63.521, I63.522, I63.523, I63.529, I63.531, I63.532, I63.533, I63.539, I63.541, I63.542, I63.543, I63.549, I63.59, I63.6, I63.8, I63.9, I67.841, I67.848, I67.89, I74.01, I74.09, I74.10, I74.11, I74.19, I74.2, I74.3, I74.4, I74.5, I74.8, I74.9, T80.0XXA, T81.718A, T81.72XA, T82.817A, T82.818A |
| Proarrhythmia hospitalization | Defined by any inpatient encounter with a diagnosis or procedure code in the principal position for one of the conditions defined below (ventricular fibrillation and flutter, ventricular tachycardia, torsades de Pointes, re-entry ventricular arrhythmia, cardiac arrest, bradycardia, sick sinus syndrome, atrioventricular block, 3rd and 2nd degree, pacemaker implantation) | |
| Ventricular fibrillation and flutter | Any inpatient encounter with an *ICD-9-CM* diagnosis code of 427.41 or 427.42 measured in the primary diagnosis position | Any inpatient encounter with an *ICD-10-CM* diagnosis code of I49.0* measured in the primary diagnosis position |
| Ventricular tachycardia | Any inpatient encounter with an *ICD-9-CM* diagnosis code of 427.1 measured in the primary diagnosis position | Any inpatient encounter with an *ICD-10-CM* diagnosis code of I47.2 measured in the primary diagnosis position |
| Torsades de Pointes | Any inpatient encounter with an *ICD-9-CM* diagnosis code of 426.82 measured in the primary diagnosis position | Any inpatient encounter with an *ICD-10-CM* diagnosis code of I45.81 or I47.2 measured in the primary diagnosis position |
| Re-entry ventricular arrhythmia | Any inpatient encounter with an *ICD-9-CM* diagnosis code of 427.1 measured in the primary diagnosis position | Any inpatient encounter with an *ICD-10-CM* diagnosis code of I47.0 measured in the primary diagnosis position |
| Cardiac arrest | Any inpatient encounter with an *ICD-9-CM* diagnosis code of 427.5 measured in the primary diagnosis position | Any inpatient encounter with an *ICD-10-CM* diagnosis code of I46.* measured in the primary diagnosis position |
| Bradycardia | Any inpatient encounter with an *ICD-9-CM* diagnosis code of 427.8* measured in the primary diagnosis position | Any inpatient encounter with an *ICD-10-CM* diagnosis code of R00.1 measured in the primary diagnosis position |
| Sick sinus syndrome | Any inpatient encounter with an *ICD-9-CM* diagnosis code of 427.81 measured in the primary diagnosis position | Any inpatient encounter with an *ICD-10-CM* diagnosis code of I49.5 measured in the primary diagnosis position |
| AV block, 3rd and 2nd degree | Any inpatient encounter with an *ICD-9-CM* diagnosis code of 426.0 or 426.13 measured in the primary diagnosis position | Any inpatient encounter with an *ICD-10-CM* diagnosis code of I44.1 or I44.2 measured in the primary diagnosis position |
| Pacemaker implantation (Proarrhythmia component) | Any inpatient encounter with any of the following procedure codes in the principal position:  *ICD-9-CM*: 00.50, 00.52, 00.53, 37.78, 37.81, 37.82, 37.83  *CPT/HCPCS*: 0387T, 33206, 33207, 33208, 33212, 33213, 33214, 33221, 33227 | Any inpatient encounter with any one of the following procedure codes in the principal position:  *ICD-10-CM*: 0JH604Z, 0JH605Z, 0JH606Z, 0JH607Z, 0JH634Z, 0JH635Z, 0JH636Z, 0JH637Z, 0JH63PZ, 0JH804Z, 0JH805Z, 0JH806Z, 0JH807Z, 0JH80PZ, 0JH834Z, 0JH835Z, 0JH836Z, 0JH837Z, 0JH83PZ, 0JH605Z  *CPT/HCPCS*: 0387T, 33206, 33207, 33208, 33212, 33213, 33214, 33221, 33227 |
| Atrial tachyarrhythmia-related procedure | Defined by its components below (screening and monitoring, cardioversion, catheter ablation, ICD*/*CRT-D insertion, pacemaker implantation) | |
| Screening and monitoring | Defined by its components below (ECG, Holter monitor/continuous ECG monitoring, exercise stress test, spirometry, echocardiogram, cardiac imaging, implantable loop recorder) | |
| Electrocardiogram | Any inpatient or ambulatory encounter with ≥1 of the following codes in any claim line position:  *ICD-9-CM* procedure: 89.51, 89.52  CPT: 0295T, 0296T, 0297T, 0298T, 93000, 93005, 93010, 93040, 93041, 93042, 93228, 93229, 93268, 93270, 93271, 93272, 93278 | Any inpatient or ambulatory encounter with ≥1 of the following codes in any claim line position:  *ICD-10-CM* procedure: 4A02X4A, 4A02X4Z, 4A02XFZ  CPT: 0295T, 0296T, 0297T, 0298T, 93000, 93005, 93010, 93040, 93041, 93042, 93228, 93229, 93268, 93270, 93271, 93272, 93278 |
| Holter monitor/continuous electrocardiogram monitoring | Any inpatient or ambulatory encounter with ≥1 of the following codes in any claim line position:  *ICD-9-CM* procedure: 89.50, 89.54  *CPT*: 93224, 93225, 93226, 93227, 93228, 93229, 93268, 93269, 93270, 93271, 93272 | Any inpatient or ambulatory encounter with ≥1 of the following codes in any claim line position:  *ICD-10-CM* procedure: 4A12X45, 4A12X4Z, 4A12XFZ  *CPT*: 93224, 93225, 93226, 93227, 93228, 93229, 93268, 93269, 93270, 93271, 93272 |
| Stress test (exercise) | Any inpatient or ambulatory encounter with ≥1 of the following codes in any claim line position:  *ICD-9-CM* procedure: 89.41, 89.42, 89.43, 89.44  *CPT/HCPCS*: 93015, 93016, 93017, 93018 | Any inpatient or ambulatory encounter with ≥1 of the following codes in any claim line position:  *ICD-10-CM* procedure: 4A12XM4  *CPT/HCPCS:* 93015, 93016, 93017, 93018 |
| Spirometry | Any inpatient or ambulatory encounter with ≥1 of the following codes in any claim line position:  *CPT/HCPCS:* 3023F, 3025F, 3027F, 94010, 94014, 94015, 94016 | |
| Echocardiogram | Any inpatient or ambulatory encounter with ≥1 of the following codes in any claim line position:  *ICD-9-CM:* 00.24, 37.28, 88.72  *CPT/HCPCS:* 0439T, 76825, 76826, 76827, 76828, 93303, 93304, 93306, 93307, 93308, 93312, 93313, 93314, 93315, 93316, 93317, 93318, 93320, 93321, 93325, 93350, 93351, 93352, 93355, 93662 | Any inpatient or ambulatory encounter with ≥1 of the following codes in any claim line position:  *ICD-10-CM:* B240ZZ4, B240ZZZ, B241ZZ4, B241ZZZ, B244YZZ, B244ZZ3, B244ZZ4, B244ZZZ, B245YZZ, B245ZZ3, B245ZZ4, B245ZZZ, B246YZZ, B246ZZ3, B246ZZ4, B246ZZZ, B24BZZ3, B24BZZ4, B24BZZZ, B24CZZ4, B24CZZZ  *CPT/HCPCS:* 0439T, 76825, 76826, 76827, 76828, 93303, 93304, 93306, 93307, 93308, 93312, 93313, 93314, 93315, 93316, 93317, 93318, 93320, 93321, 93325, 93350, 93351, 93352, 93355, 93662 |
| Cardiac imaging | Any inpatient or ambulatory encounter with ≥1 of the following procedure codes in any claim line position:  *ICD-9-CM:* 87.42, 87.44, 88.92, 92.05, 87.41  *CPT/HCPCS:* 0439T, 0541T, 0542T, 75557, 75559, 75561, 75563, 75565, 75572, 78451, 78452, 78453, 78454, 78459, 78466, 78468, 78469, 78491, 78492, 78494, 78445, 78472, 78473, 78481, 78483, 78494, 78496, 71555, 71045, 71046, 71047, 71048 | Any inpatient or ambulatory encounter with ≥1 of the following procedure codes in any claim line position:  *ICD-10-CM:* B236Y0Z, B236ZZZ, C21G1ZZ, C21GDZZ, C21GSZZ, C21GYZZ, C21GZZZ, C2261ZZ, C226YZZ, C22G1ZZ, C22GDZZ, C22GKZZ, C22GSZZ, C22GYZZ, C22GZZZ, C22YYZZ, C23GKZZ, C23GMZZ, C23GQZZ, C23GRZZ, C23GYZZ, C22YYZZ, BW03ZZZ, B22600Z, B2260ZZ, B22610Z, B2261ZZ, B226Y0Z, B226YZZ, B226Z2Z, B226ZZZ  *CPT/HCPCS:* 0439T, 0541T, 0542T, 75557, 75559, 75561, 75563, 75565, 75572, 78451, 78452, 78453, 78454, 78459, 78466, 78468, 78469, 78491, 78492, 78494, 78445, 78472, 78473, 78481, 78483, 78494, 78496, 71555, 71045, 71046, 71047, 71048 |
| Implantable loop recorder | Any inpatient or ambulatory encounter with ≥1 of the following procedure codes in any claim line position:  *CPT/HCPCS:* 33282, 33285, E0616, C1764, 0650T, 33286, 93285, 93291, 93298, 93299 | Any inpatient or ambulatory encounter with ≥1 of the following procedure codes in any claim line position:  *CPT/HCPCS:* 33282, 33285, E0616, C1764, 0650T, 33286, 93285, 93291, 93298, 93299 |
| Cardioversion | Any inpatient or ambulatory encounter with any of the following procedure codes in any claim line position:  *ICD-9-CM* procedure: 99.61, 99.62  *CPT*: 92960, 92961 | Any inpatient or ambulatory encounter with any of the following procedure codes in any claim line position:  *ICD-10-CM* procedure: 5A2204Z  *CPT*: 92960, 92961 |
| Catheter ablation | Any inpatient or ambulatory encounter with any of the following procedure codes in any claim line position: *CPT*: 93656, 93657 | Any inpatient or ambulatory encounter with any of the following procedure codes in any claim line position:  *CPT*: 93656, 93657 |
| Other rhythm control therapy | Defined by its components below: (pacemaker implantation, ICD/CRT-D insertion) | Defined by its components below: (pacemaker implantation, ICD/CRT-*D* insertion) |
| ICD/CRT-D insertion | Any inpatient or ambulatory encounter with ≥1 of the following procedure codes in any claim line position:  *ICD-9-CM:* 37.94, 00.51, 00.54  *CPT/HCPCS:* 33230, 33231, 33240, 33249, 33270 | Any inpatient or ambulatory encounter with ≥1 of the following procedure codes in any claim line position:  *ICD-10-CM:* 0JH608Z, 0JH609Z, 0JH638Z, 0JH808Z, 0JH838Z, 0JH639Z, 0JH809Z, 0JH839Z  *CPT/HCPCS*: 33230, 33231, 33240, 33249, 33270 |
| Pacemaker implantation | Any inpatient or ambulatory encounter with ≥1 of the following procedure codes in any claim line position:  *ICD-9-CM*: 00.50, 00.52, 00.53, 37.78, 37.81, 37.82, 37.83  *CPT/HCPCS*: 0387T, 33206, 33207, 33208, 33212, 33213, 33214, 33221, 33227 | Any inpatient or ambulatory encounter with ≥1 of the following procedure codes in any claim line position:  *ICD-10-CM*: 0JH604Z, 0JH605Z, 0JH606Z,  0JH607Z, 0JH634Z, 0JH635Z, 0JH636Z, 0JH637Z, 0JH63PZ, 0JH804Z, 0JH805Z, 0JH806Z, 0JH807Z, 0JH80PZ, 0JH834Z, 0JH835Z, 0JH836Z, 0JH837Z, 0JH83PZ, 0JH605Z  *CPT/HCPCS*: 0387T, 33206, 33207, 33208, 33212, 33213, 33214, 33221, 33227 |
| AF-related medication | Defined by its components below (DOACs, rate control medications, antihypertensives, AADs) | |
| Anticoagulants (time since initiation until index date) | Continuous, days  Among patients who have a claim for oral anticoagulants (defined above) during baseline, the number of days from the earliest prescription claim during the baseline period to the index date (exclusive) | |
| Warfarin | The occurrence of any prescription claim with the generic name “Warfarin Sodium”; mean daily dose of prescriptions filled during baseline, defined as [(strength*quantity)/days’ supply] | |
| DOACs | The occurrence of any prescription claim with the following generic names: “Apixaban”, “Edoxaban Tosylate”, “Dabigatran Etexilate Mesylate”, “Rivaroxaban” as mean daily dose of prescriptions filled during baseline, defined as [(strength*quantity)/days’ supply] | |
| Digoxin | Any prescription claim with the generic name “digoxin” or the “C01AA05” WHO ATC code prefix | |
| P2Y12 agent | Any prescription claim with the following generic names: “clopidogrel,” “ticlopidine,” “ticagrelor,” or “prasugrel” | |
| Rate control medications | Defined as (beta-blocking agents, calcium channel blockers) | |
| Beta-blocking agents | Any prescription claim with the WHO ATC code prefix: C07 | |
| Calcium-channel blockers (DHP or NDHP) | Any prescription claim with any of the following WHO ATC code prefixes: C07FB, C08, C09BB, C09DB, C10BX03, C10BX07, C10BX09, C10BX11, C10BX14, C10BX18 | |
| Antihypertensives | Defined as (angiotensin-converting enzyme inhibitors, angiotensin-II receptor blockers, aldosterone, diuretics) | |
| Angiotensin-converting enzyme inhibitors | Any prescription claim with any of the following WHO ATC code prefixes: C09A, C09B, C10BX04, C10BX06, C10BX07, C10BX11, C10BX12, C10BX13, C10BX14, C10BX15, C10BX17, C10BX18 | |
| Angiotensin-II receptor blockers | Any prescription claim with the following WHO ATC code prefixes: C09C, C09D, C10BX10, C10BX16 | |
| Aldosterone | Any prescription claim with the “C03DA” WHO ATC code prefix | |
| Diuretics | Any prescription claim with any of the following WHO ATC code prefixes: C02L, C03, C07B, C07C, C07D, C08GA, C09BA, C09DA, C10BX13 | |
| AADs at index in first-line ablation patients indexing on AAD therapy (other than dronedarone) | The occurrence of any prescription claim with the following generic names:  “Amiodarone,” “Sotalol,” “Flecainide,” “Propafenone,” or “Dofetilide” as: mean daily dose of prescriptions filled during baseline, defined as [(strength*quantity)/days supply] | |
| AAD, antiarrhythmic drug; AF, atrial fibrillation; ATC, Anatomical Therapeutic Chemical; CRT-D, cardiac resynchronization therapy with defibrillator; DOAC, direct acting oral anticoagulant; DHP, dihydropyridine; *ICD-9-CM, International Classification of Diseases, Ninth Revision, Clinical Modification*; *ICD-10-CM, International Classification of Diseases, Tenth Revision, Clinical Modification*; NDHP, non-dihydropyridine; TE, thromboembolism; TIA, transient ischemic attack; WHO, World Health Organization. | | |

**TABLE S3 Variables used in the propensity score matching.**

| **Variable** | **Classification/Definition** |
| --- | --- |
| Age at index (years; rounded to integer) | Continuous and categorical |
| Sex | Male, Female (categorical) |
| Region | Northeast, North Central, South, West (categorical) |
| Payer | Commercial, Medicare, None, Unknown (categorical) |
| Health plan type | National Ancillaries Contracted for All Products, EPO, HMO, Indemnity, POS, PPO, None, Other (categorical) |
| Year of index date | Categorical |
| Time from start of available data to index date (days) | Categorical |
| Comorbidities | |
| Diabetes | Yes/No (binary) |
| Heart failure | Yes/No (binary) |
| Hypertension | Yes/No (binary) |
| Obstructive sleep apnea | Yes/No (binary) |
| Obesity | Yes/No (binary) |
| Mitral stenosis | Yes/No (binary) |
| Pulmonary hypertension | Yes/No (binary) |
| Stroke, TIA or TE | Yes/No (binary) |
| Vascular disease | Yes/No (binary) |
| Atrial flutter | Yes/No (binary) |
| Time from AF diagnosis to index date | Yes/No (binary) |
| CCI^a^ | Continuous and categorical (0, 1–2, 3–4, 5+) |
| CHA_2_DS_2_-VASc score^a^ | Continuous and categorical low risk (score = 0), intermediate risk (score = 1), high risk (scores = 2–9) |
| Baseline therapies |  |
| Cardioversion | Yes/No (Binary) |
| Any DOACs | Yes/No (Binary) |
| Warfarin | Yes/No (Binary) |
| Antiplatelet therapy (P2Y12 inhibitors) | Yes/No (Binary) |
| Rate control medication (beta blockers, CCB) | Yes/No (Binary) |
| Digoxin | Yes/No (Binary) |
| Antihypertensives (ACE inhibitors, ARBs, aldosterone antagonists, diuretics) | Yes/No (Binary) |
| Baseline HCRU | |
| Number of all-cause outpatient visits (any) | Continuous |
| Number of all-cause outpatient physician office visits | Continuous |
| Number of all-cause ER visits | Continuous |
| Number of days hospitalized (all-cause) in the inpatient setting | Continuous |
| ^a^For CCI and CHA_2_DS_2_-VASc scores, patients were matched on select score components, not on the scores themselves.  ACE, angiotensin converting enzyme; AF, atrial fibrillation; ARB, angiotensin receptor blocker;  CCB, calcium channel blocker; CCI, Charlson Comorbidity Index; DOAC, direct-acting oral anticoagulant; EPO, exclusive provider organization; ER, emergency room; HCRU, healthcare resource utilization; HMO, health maintenance organization; PPO, preferred provider organization; POS, point of service; TE, thromboembolism; TIA, transient ischemic attack. | |

**TABLE S4 Number of patients with AF taking AAD in the main cohort analysis of propensity score matched dronedarone^a^ and first-line ablation patients.**

|  | **PSM cohort^b^** | |
| --- | --- | --- |
|  | **First-line dronedarone  (*N* = 1440)** | **First-line ablation  (*N* = 1440)** |
| **Any AAD^a^** |  | |
| 3 months, *n* (%) | 1440 (100) | 789 (54.8) |
| 6 months, *n* (%) | 1440 (100) | 806 (56.0) |
| 12 months, *n* (%) | 1440 (100) | 826 (57.4) |
| 24 months, *n* (%) | 1440 (100) | 838 (58.2) |
| ^a^Dronedarone arm includes patients taking dronedarone as part of the study inclusion criteria. ^b^Variables used in propensity score matching are given in Table S3.  AAD, antiarrhythmic drug; AF, atrial fibrillation. | | |

**TABLE S5 HCRU event count and event rate ratios (95% CI) for patients with AF treated with first-line dronedarone versus first-line ablation in a sensitivity analysis of patients with at least 12 months of follow-up.**

|  | **PSM cohort^a^** | | ***p*-value** |
| --- | --- | --- | --- |
|  | **First-line dronedarone**  **(*n* = 1212)** | **First-line ablation**  **(*n* = 1212)** |  |
| Inpatient (hospital stay) |  |  |  |
| Any event, *n* (%) | 265 (21.9) | 360 (29.7) | < .01 |
| Total events, mean ± SD | 0.3 ± 0.9 | 0.5 ± 0.9 | < .01 |
| ERR (95% CI)^b^ | 0.77 (0.67–0.87) | | < .01 |
| Outpatient – any visits | | | |
| Any event, *n* (%) | 1211 (99.9) | 1212 (100.0) | 1.00 |
| Total events, mean ± SD | 28.9 ± 23.7 | 31.7 ± 20.6 | < .01 |
| ERR (95% CI)^b^ | 0.91 (0.90–0.93) | | < .01 |
| Outpatient - physician office visits | | | |
| Any event, *n* (%) | 1208 (99.7) | 1210 (99.8) | .68 |
| Total events, mean ± SD | 12.0 ± 7.7 | 13.0 ± 7.6 | < .01 |
| ERR (95% CI)^b^ | 0.92 (0.90–0.94) | | < .01 |
| Outpatient – ER visits |  |  |  |
| Any event, *n* (%) | 443 (36.6) | 492 (40.6) | .05 |
| Total events, mean ± SD | 0.71 ± 1.62 | 0.83 ± 1.86 | .1 |
| ERR (95% CI)^b^ | 0.86 (0.79–0.94) | | < .01 |
| ^a^Variables used in PSM are given in Table S3. ^b^Poisson regression models were used to estimate PSM-adjusted ERRs and 95% CIs for HCRU count outcomes. HCRU was recorded at any time after index. Median follow-up was 366 days in both first-line dronedarone and ablation cohorts.  AF, atrial fibrillation; ER, emergency room; ERR, event rate ratio; HCRU, healthcare resource utilization; PSM, propensity score matched. | | | |

**TABLE S6 Associated PPPM costs (in $) for all-cause HCRU for patients with AF treated with first-line dronedarone versus first-line ablation in a sensitivity analysis of patients with at least 12 months of follow-up.**

| **PPPM Costs, $** | **PSM cohort^a^** | | **Incremental PPPM Cost, $** |  |
| --- | --- | --- | --- | --- |
|  | **First-line dronedarone**  **(*N* = 1212)** | **First-line ablation**  **(*N* = 1212)** |  | ***p*-value**^b^ |
| Total payer costs | | | | |
| Mean ± SD | 2799 ± 6,792 | 6153 ± 4839 | 3354 | < .01 |
| Median  [min, max] | 813  [0, 127,684] | 5108  [82, 47,709] |  |  |
| Inpatient visits (hospital stays) | | | | |
| Mean ± SD | 682 ± 2273 | 940 ± 2497 | 258 | .21 |
| Median  [min, max] | 0  [0, 27,657] | 0  [0, 19,748] |  |  |
| Outpatient – any visits | | | | |
| Mean ± SD | 2117 ± 5983 | 5213 ± 4179 | 3096 | < .01 |
| Median  [min, max] | 708 [0, 127,684] | 4815  [11.5, 47,709] |  |  |
| Outpatient - physician office visits | | | | |
| Mean ± SD | 95.5 ± 69.4 | 105 ± 70.9 | 9.1 | < .01 |
| Median  [min, max] | 79.7  [0, 552] | 89.2  [0, 604] |  |  |
| Outpatient – ER visits | | | | |
| Mean ± SD | 89.5 ± 214 | 105 ± 267 | 15.3 | .43 |
| Median  [min, max] | 0  [0, 2518] | 0  [0, 5191] |  |  |
| ^a^Variables used in PSM are given in Table S3. ^b^*p*-values for the estimate of marginal cost difference were estimated using PSM-adjusted zero-inflated negative binomial regression.  AF, atrial fibrillation; ER, emergency room; HCRU, healthcare resource utilization; PSM, propensity score matched. | | | | |

**TABLE S7 Select baseline^a^ demographics, characteristics, HCRU, and procedures for patients with AF after PSM in a sensitivity analysis where ablation patients had no prior AAD use (including within 90 days of ablation).**

| **Characteristic**  **Data mean ± SD or *n* (%)** | **After PSM** | | **ASD**^b^ |
| --- | --- | --- | --- |
|  | **First-line dronedarone  (*N* = 1120)** | **First-line  ablation only (*N* = 1120)** |  |
| Age at index,^c^ y | 68.2 ± 10.7 | 68.2 ± 9.5 | 0.00 |
| **Sex**^c^ | | | |
| Male | 672 (60.0) | 679 (60.6) | 0.01 |
| Female | 448 (40.0) | 441 (39.4) |  |
| **Region**^c^ | | | |
| Midwest | 228 (20.4) | 243 (21.7) | 0.06 |
| South | 519 (46.3) | 525 (46.9) |  |
| West | 240 (21.4) | 224 (20.0) |  |
| Northeast | 132 (11.8) | 125 (11.2) |  |
| Other/Unknown | 1 (0.1) | 3 (0.3) |  |
| **Payer**^c^ |  |  | 0.00 |
| Commercial | 394 (35.2) | 393 (35.1) |  |
| Medicare Advantage | 726 (64.8) | 727 (64.9) |  |
| Time from AF diagnosis to index date, days^c^ | 94.8 ± 103.1 | 100.5 ± 78.4 |  |
| **Baseline comorbidities^a^** |  |  | 0.00 |
| CHA_2_DS_2_-VASc score^c,d^ | 3.3 (1.8) | 3.3 (1.8) |  |
| CHA_2_DS_2_-VASc category^c,d^ |  |  | 0.05 |
| Low risk (score: 0) | 41 (3.7) | 52 (4.6) |  |
| Intermediate risk  (score: 1) | 143 (12.8) | 142 (12.7) |  |
| High risk (score: ≥2) | 936 (83.6) | 926 (82.7) |  |
| CCI,^c,d^ mean ± SD | 2.3 ± 2.2 | 2.4 ± 2.3 | 0.04 |
| CCI category^c,d^ |  |  | 0.08 |
| 0 | 256 (22.9) | 272 (24.3) |  |
| 1–2 | 451 (40.3) | 418 (37.3) |  |
| 3–4 | 246 (22.0) | 240 (21.4) |  |
| ≥5 | 167 (14.9) | 190 (17.0) |  |
| **History of comorbidities** |  |  |  |
| Hypertension^c^ | 917 (81.9) | 934 (83.4) | 0.04 |
| CAD | 817 (72.9) | 866 (77.3) | 0.10 |
| Vascular disease^c^ | 393 (35.1) | 380 (33.9) | 0.02 |
| Obesity^c^ | 360 (32.1) | 376 (33.6) | 0.03 |
| Heart failure^c^ | 262 (23.4) | 271 (24.2) | 0.02 |
| Hypothyroidism | 244 (21.8) | 238 (21.3) | 0.01 |
| Obstructive sleep apnea^c^ | 246 (22.0) | 266 (23.8) | 0.04 |
| CKD | 224 (20.0) | 238 (21.3) | 0.03 |
| COPD | 192 (17.1) | 164 (14.6) | 0.07 |
| Stroke, TIA or TE^c^ | 125 (11.2) | 134 (12.0) | 0.03 |
| Peripheral artery disease | 111 (9.9) | 83 (7.4) | 0.09 |
| Myocardial infarction | 76 (6.8) | 69 (6.2) | 0.03 |
| Pulmonary hypertension^c^ | 70 (6.3) | 70 (6.3) | 0.00 |
| Venous thromboembolism | 42 (3.8) | 29 (2.6) | 0.07 |
| Hyperthyroidism | 31 (2.8) | 14 (1.3) | 0.11 |
| Mitral stenosis^c^ | 14 (1.3) | 7 (0.6) | 0.07 |
| **Baseline medication use** |  |  |  |
| Any DOAC^c^ | 609 (54.4) | 608 (54.3) | 0.00 |
| Warfarin^c^ | 101 (9.0) | 104 (9.3) | 0.01 |
| Digoxin^c^ | 60 (5.4) | 68 (6.1) | 0.03 |
| P2Y12 agent^c^ | 102 (9.1) | 98 (8.8) | 0.01 |
| Any rate control medication^c^ | 836 (74.6) | 848 (75.7) | 0.03 |
| Any antihypertensive^c^ | 665 (59.4) | 672 (60.0) | 0.01 |
| **Baseline procedures^a^** |  |  |  |
| Cardioversion^c^ | 315 (28.1) | 329 (29.4) | 0.03 |
| ATA screening and monitoring | 1108 (98.9) | 1118 (99.8) | 0.11 |
| **HCRU and costs,**^e^ **$** |  |  |  |
| Inpatient days^c^ | 2.8 ± 5.6 | 2.6 ± 5.2 | 0.03 |
| Any outpatient visits^c^ | 24.3 ± 22.0 | 25.1 ± 17.9 | 0.04 |
| Outpatient – physician office visits^c^ | 10.4 ± 7.4 | 10.5 ± 6.1 | 0.02 |
| Total medical and prescription payer costs,^e^ $ | 31,667 ± 51,811 | 29,965 ± 47,362 | 0.03 |
| ^a^Demographic characteristics were assessed at index, and baseline comorbidities and procedures were assessed during the baseline period, defined as 365 days prior to 1 day before index. ^b^A covariate was considered balanced after PSM if ASD was ≤ 0.1 (10%) between treatment groups. ^c^Included in the propensity score model. ^d^CHA_2_DS_2_-VASc and CCI scores calculated during baseline period were both categorical and continuous. For PSM, patients were matched on individual components not on the scores themselves. ^e^All costs were adjusted to 2020$ using the Standard Cost Amount field and Optum’s Cost Factors so costs during the study period could be directly compared. Total costs were calculated as the sum of inpatient payer costs and any outpatient visit payer costs.  AAD, antiarrhythmic drug; AF, atrial fibrillation; ASD, absolute standardized difference; ATA, atrial tachyarrhythmia; CAD, coronary artery disease; CCI, Charlson Comorbidity Index; CKD, chronic kidney disease; COPD, chronic obstructive pulmonary disease; DOAC, direct-acting oral anticoagulant; HCRU, healthcare resource utilization; PSM, propensity score matching; TE, thromboembolism; TIA, transient ischemic attack. | | | |

**TABLE S8 HCRU event count and event rate ratios (95% CI) for patients with AF treated with first-line dronedarone versus first-line ablation, in a sensitivity analysis of patients where ablation patients had no prior AAD use (including within 90 days of ablation).**

|  | **PSM cohort^a^** | | ***p*-value** |
| --- | --- | --- | --- |
|  | **First-line dronedarone**  **(*n* = 1120)** | **First-line ablation**  **(*n* = 1120)** |  |
| Follow-up days  Median [interquartile range] | 610.4 ± 183.0  731 [487–731] | 609.8 ± 184.8  731 [491–731] | .94 |
| Inpatient visits (hospital stays) |  |  |  |
| Any event, *n* (%) | 322 (28.8) | 384 (34.3) | < .01 |
| Total events, mean ± SD | 0.6 ± 1.2 | 0.6 ± 1.1 | .69 |
| ERR (95% CI)^b^ | 0.96 (0.86–1.08) | | .52 |
| Outpatient – any visits | | | |
| Any event, *n* (%) | 1120 (100) | 1120 (100) | – |
| Total events, mean ± SD | 45.6 ± 39.6 | 45.5 ± 36.2 | .91 |
| ERR (95% CI)^b^ | 1.00 (0.99–1.02) | | .63 |
| Outpatient – physician office visits | | | |
| Any event, *n* (%) | 1116 (99.6) | 1114 (99.5) | .75 |
| Total events, mean ± SD | 18.4 ± 12.6 | 17.9 ± 11.9 | .35 |
| ERR (95% CI)^b^ | 1.03 (1.01–1.05) | | < .01 |
| Outpatient – ER visits |  |  |  |
| Any event, *n* (%) | 521 (46.5) | 529 (47.2) | .77 |
| Total events, mean ± SD | 1.1 ± 2.0 | 1.2 ± 2.9 | .37 |
| ERR (95% CI)^b^ | 0.92 (0.85–1.00) | | .04 |
| ^a^Variables used in PSM are given in Table S3. Baseline demographics and clinical characteristics were similar to the primary cohort (see Table 1: mean age 68.2 years; ~60% male; mean CHA_2_DS_2_-VASc score: 3.3). ^b^Poisson regression models were used to estimate PSM-adjusted ERRs and  95% CIs for HCRU count outcomes. HCRU was recorded at any time on or after index.  AAD, antiarrhythmic drug; AF, atrial fibrillation; ER, emergency room; ERR, event rate ratio; HCRU, healthcare resource utilization; PSM, propensity score matched. | | | |

**TABLE S9 Associated PPPM costs (in $) for all-cause HCRU for patients with AF treated with first-line dronedarone vs first-line ablation, in a sensitivity analysis of patients where ablation patients had no prior AAD use (including within 90 days of ablation).**

| **PPPM costs, $** | **PSM cohort^a^** | | **Incremental PPPM cost, $** |  |
| --- | --- | --- | --- | --- |
|  | **First-line dronedarone**  **(*N* = 1120)** | **First-line ablation**  **(*N* = 1120)** |  | ***p*-value**^b^ |
| Total payer costs | | | | |
| Mean ± SD | 2968 ± 6979 | 5070 ± 6402 | 2102.0 | <0.01 |
| Median  [min, max] | 960  [8.9, 112,694] | 3447  [54.1, 72,259] | — | — |
| Inpatient visits (hospital stays) | | | | |
| Mean ± SD | 883.3±3,333 | 988.7±3,542 | 105.4 | 0.96 |
| Median  [min, max] | 0  [0, 62,073] | 0  [0, 55,190] | — | — |
| Outpatient – any visits | | | | |
| Mean ± SD | 2084.5 ±  5362.4 | 4081.0 ± 4532.2 | 1996.6 | <0.01 |
| Median  [min, max] | 773.9  [8.9, 112,694] | 2,908.5  [16.8, 51,432] | — | — |
| Outpatient – physician office visits | | | | |
| Mean ± SD | 90.5 ± 67.5 | 86.9 ± 61.7 | −3.6 | 0.19 |
| Median  [min, max] | 74.5  [0, 585.1] | 73.6  [0, 611.7] | — | — |
| Outpatient – ER visits | | | | |
| Mean ± SD | 89.1 ± 190.5 | 90.2 ± 224.1 | 1.1 | 0.98 |
| Median  [min, max] | 0  [0, 2,471] | 0  [0 4,143] | — | — |
| ^a^Variables used in PSM are given in Table S3**.** ^b^*p*-values for the estimate of marginal cost difference were estimated using PSM-adjusted zero-inflated negative binomial regression.  AAD, antiarrhythmic drug; AF, atrial fibrillation; ER, emergency room; HCRU, healthcare resource utilization; PPPM, per-patient per-month; PSM, propensity score matched. | | | | |
